# The Single‐Cell Landscape of Intratumoral Heterogeneity and The Immunosuppressive Microenvironment in Liver and Brain Metastases of Breast Cancer

**DOI:** 10.1002/advs.202203699

**Published:** 2022-12-18

**Authors:** Yutian Zou, Feng Ye, Yanan Kong, Xiaoqian Hu, Xinpei Deng, Jindong Xie, Cailu Song, Xueqi Ou, Song Wu, Linyu Wu, Yi Xie, Wenwen Tian, Yuhui Tang, Chau‐Wei Wong, Zhe‐Sheng Chen, Xinhua Xie, Hailin Tang

**Affiliations:** ^1^ Sun Yat‐sen University Cancer Center State Key Laboratory of Oncology in South China Collaborative Innovation Center for Cancer Medicine 651 East Dongfeng Road Guangzhou 510060 China; ^2^ School of Biomedical Sciences Faculty of Medicine The University of Hong Kong 21 Sassoon Road Hong Kong 999077 China; ^3^ College of Pharmacy and Health Sciences St. John's University Queens NY USA

**Keywords:** breast cancer, immune checkpoint, metastatic niche, single‐cell RNA sequencing, tumor microenvironment

## Abstract

Distant metastasis remains the major cause of morbidity for breast cancer. Individuals with liver or brain metastasis have an extremely poor prognosis and low response rates to anti‐PD‐1/L1 immune checkpoint therapy compared to those with metastasis at other sites. Therefore, it is urgent to investigate the underlying mechanism of anti‐PD‐1/L1 resistance and develop more effective immunotherapy strategies for these patients. Using single‐cell RNA sequencing, a high‐resolution map of the entire tumor ecosystem based on 44 473 cells from breast cancer liver and brain metastases is depicted. Identified by canonical markers and confirmed by multiplex immunofluorescent staining, the metastatic ecosystem features remarkable reprogramming of immunosuppressive cells such as FOXP3+ regulatory T cells, LAMP3+ tolerogenic dendritic cells, CCL18+ M2‐like macrophages, RGS5+ cancer‐associated fibroblasts, and LGALS1+ microglial cells. In addition, PD‐1 and PD‐L1/2 are barely expressed in CD8+ T cells and cancer/immune/stromal cells, respectively. Interactions of the immune checkpoint molecules LAG3‐LGALS3 and TIGIT‐NECTIN2 between CD8+ T cells and cancer/immune/stromal cells are found to play dominant roles in the immune escape. In summary, this study dissects the intratumoral heterogeneity and immunosuppressive microenvironment in liver and brain metastases of breast cancer for the first time, providing insights into the most appropriate immunotherapy strategies for these patients.

## Graphical abstract

High‐resolution landscapes of the entire tumor ecosystem from breast cancer liver and brain metastases are first depicted using single‐cell RNA sequencing. The metastatic breast cancer microenvironment features remarkable intratumoral heterogeneity and abundant immunosuppressive cells. This study provides unique insights into the immunotherapeutic strategies most appropriate for patients with liver or brain metastatic breast cancer.

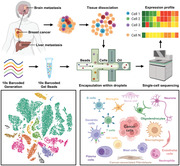

## 1 Introduction

According to the latest global statistics, breast cancer has become the most common cancer throughout the world, with an estimated 2.3 million new cases in 2020.^[^ ^1^ ^]^ Although most patients with early breast cancer can be cured, a considerable number of patients (≈20–30%) will still experience local recurrence or distant metastasis within two years of diagnosis of the primary tumor, and these outcomes are a major cause of morbidity for breast cancer patients.^[^ ^2^ ^]^ The 5‐year overall survival rate of patients with nonmetastatic breast cancer is greater than 80%, while that of patients with distant metastasis is less than 25%.^[^ ^3^ ^]^ Among patients with metastatic breast cancer, patients with liver or brain metastasis have an extremely high progression rate, poor prognosis, and low quality of life.^[^ ^4^ ^]^ In the past decade, targeting immune checkpoints has become an effective therapeutic strategy for various metastatic solid malignant tumors. Despite the promising clinical progress of immune checkpoint therapy in treating metastatic breast cancer, only a very small percentage of patients benefit from it, and adverse events frequently occur.^[^ ^5^ ^]^ According to the subgroup analyses of several clinical trials, breast cancer patients with liver or brain metastasis have a low response rate to anti‐PD‐1/L1 immune checkpoint therapy compared with patients with metastasis at other sites.^[^ ^5^, ^6^, ^7^ ^]^ Therefore, it is urgent to investigate the fundamental mechanism of anti‐PD‐1/L1 immune checkpoint therapy resistance and develop more effective immunotherapy strategies for liver and brain metastasis of breast cancer.

The tumor microenvironment is a self‐regulating ecosystem composed of tumor cells, stromal cells, and immune cells interwoven with noncellular components. Multifarious cellular phenotypes, as well as the dynamic communication between these tumor microenvironment members, shape the organ‐specific tumor milieu and may contribute to different responses to immune checkpoint therapy.^[^ ^8^, ^9^ ^]^ Traditional bulk RNA sequencing has low resolution and provides limited data to analyze microenvironmental components due to its inability to distinguish the RNA profiles of individual cells. In recent years, single‐cell RNA sequencing (scRNA‐seq) has been widely used in the study of tumor cell heterogeneity, the discovery of new mutation sites, the study of cloning and evolution mechanisms of tumor cells, and the identification of new therapeutic targets.^[^ ^10^, ^11^ ^]^ Additionally, scRNA‐seq is a powerful tool for researchers to analyze the heterogeneity of tumor‐infiltrating immune cells and their interactions with different cell types in the tumor ecosystem.^[^ ^12^, ^13^, ^14^, ^15^, ^16^ ^]^ Several studies have dissected the heterogeneity in different subtypes of primary breast cancer and identified cell clusters associated with poor prognosis or treatment response. Wu et al. analyzed 26 primary breast cancer tissues and developed an scRNA‐seq method of intrinsic subtype classification (SCSubtype) to elucidate the cellular architecture of the tumor microenvironment. Based on the proportion and spatial relationship of each cell subcluster, nine ecotypes of primary breast cancer were identified and proven to be associated with prognosis.^[^ ^17^ ^]^ By comparing the changes in the immune microenvironment between primary breast cancer with and without T cell clonal proliferation after treatment, Bassez et al. revealed the differentiation trajectory of various immune cells in response to immunotherapy and the possible mechanism of activation.^[^ ^18^ ^]^ They also found that the main cell types expressing PD‐L1 in breast cancer were not tumor cells but macrophages and dendritic cells, and the expression of PD‐L1 in the latter two cell types could predict the immunotherapy response.^[^ ^18^ ^]^ Zhang et al. utilized scRNA‐seq and scATAC‐seq methods to investigate immune cell dynamics in 22 patients with triple‐negative breast cancer after atezolizumab treatment. According to their results, the presence of CXCL13‐positive T cells increased the sensitivity of triple‐negative breast cancer to anti‐PD‐L1 therapy.^[^ ^19^ ^]^ Davis  et al. used primary breast cancer tissues to establish patient‐derived xenograft (PDX) models and used the scRNA‐seq method to investigate the transcriptional diversity and bioenergetic shifts of metastases.^[^ ^20^ ^]^ However, in breast cancer research, most scRNA‐seq studies have focused on the primary lesion, while studies of systematic single‐cell characterization of metastatic lesions (especially liver and brain metastases) have not been reported to date.

In this study, we dissected intratumoral heterogeneity and the immunosuppressive microenvironment in liver and brain metastases of breast cancer at single‐cell resolution. scRNA‐seq identified diverse cell types in liver and brain metastases of breast cancer, including cancer cells, lymphocytes, myeloid cells, stromal cells, and organ‐specific resident cells. In particular, the metastatic ecosystem was found to feature remarkable reprogramming of immunosuppressive cells such as FOXP3+ Treg cells, LAMP3+ tolerogenic DCs, CCL18+ M2‐like macrophages, RGS5+ cancer‐associated fibroblasts (CAFs), and LGALS1+ microglial cells. We found that the expression of the immunoreceptor inhibitory checkpoint genes *LAG3* and *TIGIT* in T cells was higher than that of *PDCD1* (*PD‐1*). Consistently, the corresponding immune checkpoint ligands *CD274* (*PD‐L1*) and *PDCD1LG2* (*PD‐L2*) were barely expressed in both cancer cells and other infiltrating immune/stromal cells in the tumor microenvironment. Other immune checkpoint ligands, such as *NECTIN2*, *LGALS3*, *LGALS9*, and *SELPLG*, were highly expressed in cancer cells or other infiltrating immune/stromal cells. These data provide insights into the immunotherapeutic strategies most appropriate for liver and brain metastatic breast cancer.

## 2 Results

### 2.1 Tumor Ecosystem of Breast Cancer Liver and Brain Metastases Characterized by Single‐Cell Transcriptomic Sequencing

**Figure 1:**
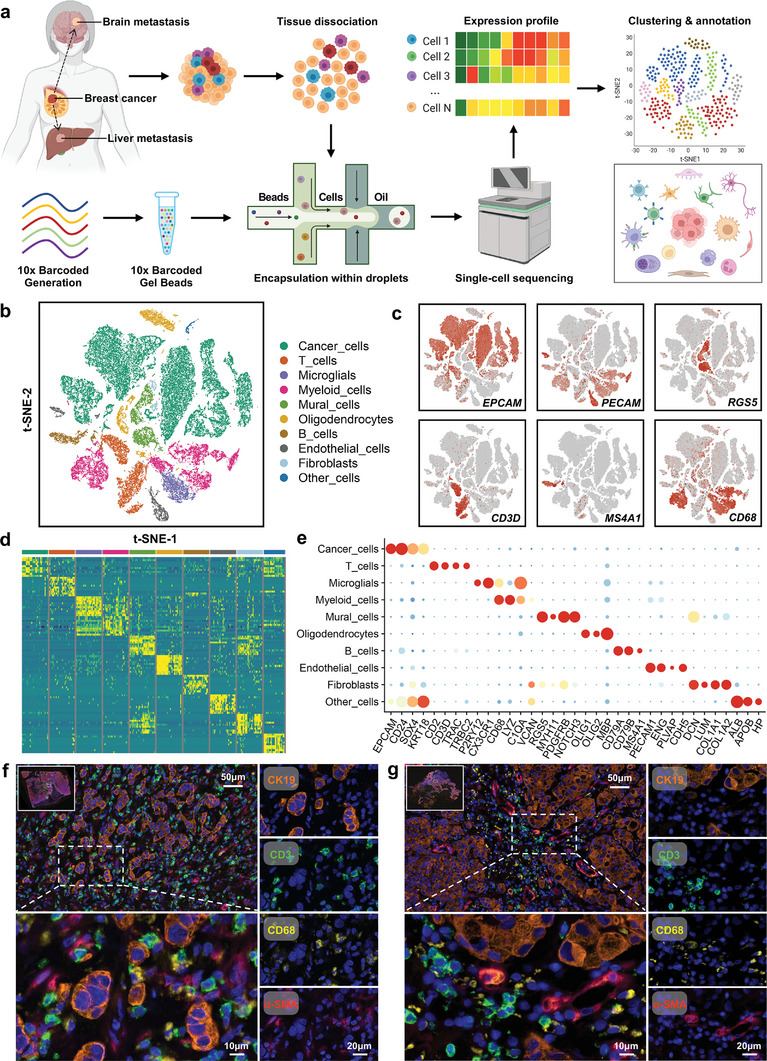
Tumor ecosystem of breast cancer liver and brain metastases characterized by single‐cell transcriptomic sequencing. a) Overview of the study design and workflow. Single‐cell suspensions were collected from liver‐ and brain‐metastatic lesions of breast cancer followed by single‐cell transcriptomic sequencing on the 10× Genomics platform. b) t‐SNE plot of single cells profiled in the present study colored by major cell type. c) Feature plots for the canonical marker genes of epithelial cancer cells (*EPCAM*), endothelial cells (*PECAM1*), mural cells (*RGS5*), T cells (*CD3D*), B cells (*MS4A1*), and myeloid cells (*CD68*). d) Heatmap of the expression levels of the top 10 differentially expressed genes among ten major cell types in liver and brain metastases of breast cancer. e) Dot plot showing the expression levels of canonical marker genes across all major cell types. f,g) Representative images of multiplex immunofluorescent staining of f) liver and g) brain metastases of breast cancer. The orange, green, yellow, and red colors indicate positive cells with the expression of CK19 (cancer cells), CD3 (T cells), CD68 (myeloid cells), and *α*‐SMA (fibroblasts and endothelial cells), respectively, in liver and brain metastases of breast cancer.

To decipher the cellular architecture within the tumor microenvironment in liver and brain metastases of breast cancer, we performed single‐cell RNA sequencing of the metastatic lesions (**Figure** **1**a). After strict quality control filters and doublet removal, a total of 44473 cells were identified and included in the subsequent analysis. We detected ≈2411 genes and 9799 unique molecular identifiers (UMIs) on average for each cell (Figure S1, Supporting Information). Then, unsupervised clustering analysis was performed in the Seurat program to define major clusters of cells with similar expression patterns (Figure 1b and Figure S2a, Supporting Information). According to the expression of canonical markers and the most variable genes, each cluster was confirmed as a specific cell subpopulation: cancer cells (gene markers: *EPCAM*, *CD24*, *SOX4*, and *KRT18*), T cells (gene markers: *CD2*, *CD3D*, *TRAC*, and *TRBC2*), B cells (gene markers: *CD79A*, *CD79B*, and *MS4A1*), myeloid cells (gene markers: *LYZ*, *MNDA*, *C1QA*, *VCAN*, and *APOE*), fibroblasts (gene markers: *COL1A1*, *COL1A2*, *LUM*, and *DCN*), mural cells (gene markers: *RGS5*, *MYH11*, *PDGFRB*, and *NOTCH3*), endothelial cells (gene markers: *PECAM1*, *ENG*, *PLVAP*, and *CDH5*), microglial cells (gene markers: *P2RY12* and *CX3CR1*), oligodendrocytes (gene markers: *OLIG1*, *OLIG2*, *MBP*, *MOG*, and *MAG*), and other cells (gene markers: *ALB*, *APOB*, and *HP*) (Figure 1c,d). We identified ten main cell populations: cancer cells (*N* = 24 187), T cells (*N* = 3885), B cells (*N* = 1178), myeloid cells (*N* = 6526), fibroblasts (*N* = 407), mural cells (*N* = 2607), endothelial cells (*N* = 1246), microglial cells (*N* = 1660), oligodendrocytes (*N* = 2532), and other cells (*N* = 245) (Figure S2b, Supporting Information). The scaled expression levels and proportions of cell expressing cluster‐specific markers in each cell subpopulation are displayed in dot plots (Figure 1e). We further performed multiplex immunofluorescent staining to confirm the cell subpopulations in the liver and brain metastasis tissues of breast cancer (Figure 1f,g). To assess the immunosuppressive microenvironment in the liver and brain metastatic lesions of breast cancer, we examined the expression of immune checkpoint molecules in each cell subpopulation (Figure S2c, Supporting Information). We found that the expression of *LAG3*, *TIGIT*, *CD96*, and *KLRB1* in T cells was higher than that of *PDCD1* (*PD‐1*) (Figure S2d, Supporting Information). Consistently, the corresponding immune checkpoint ligands *CD274* (*PD‐L1*) and *PDCD1LG2* (*PD‐L2*) were barely expressed in either cancer cells or other infiltrating cells in the tumor microenvironment (Figure S2d, Supporting Information). Other immune checkpoint ligands, *NECTIN2*, *LGALS3*, and *LGALS9* were highly expressed in cancer cells or other infiltrating cells (Figure S2d, Supporting Information). These results indicate that the PD‐1 receptor and PD‐L1/2 ligand pair may not be the dominant immune checkpoint signaling pathway molecules in liver and brain metastases of breast cancer. Therefore, targeting the LAG3‐LGALS3 and TIGIT‐NECTIN2 pairs may be an effective strategy for treating liver and brain metastases of breast cancer.

### 2.2 Identification of Six Common Expression Programs of Cancer Cells in Breast Cancer Liver and Brain Metastatic Lesions

**Figure 2:**
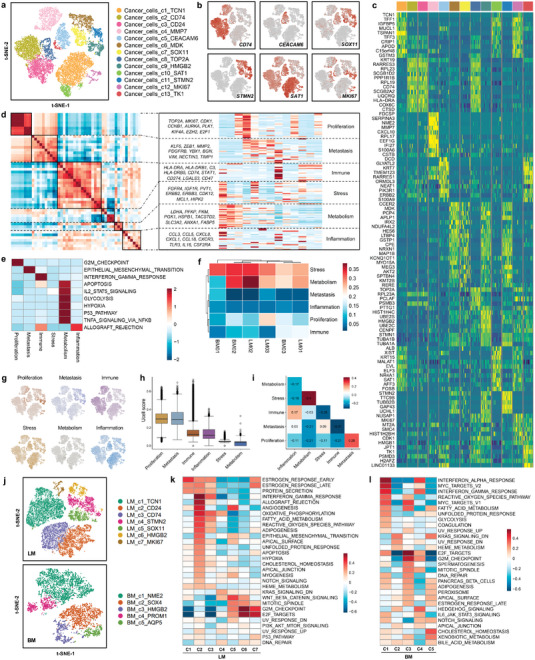
Identification of six common expression programs of cancer cells in liver‐ and brain‐metastatic breast cancer lesions. a) t‐SNE plot of the cancer cell landscape colored by cluster. b) Feature plots showing the normalized expression of highly expressed genes in each cancer cell subcluster. c) Heatmap of the expression levels of the top 10 differentially expressed genes among thirteen subclusters of cancer cells. d) Heatmap showing pairwise correlations of 50 modules derived from the cancer cell gene expression profile. Six common expression programs across cancer cells are aggregated into clusters. e) GSVA was performed to compare the differences in pathways between program cells and nonprogram cells for each program. The Z score was used to normalize each column. f) Normalized program scores for all six expression programs in each sample are shown in a clustered heatmap. g) Distributions of each expression program. Cells expressing over 70% of genes in each program were defined as program cells; otherwise, they were defined as nonprogram cells. h) Boxplot of the proportions of program cells among six expression programs, ordered by the median program score. i) Heatmap revealing the odds ratios as a measure of the co‐occurrence or exclusion of each expression program pair (columns and rows). j) Reclustering of cancer cells in liver metastasis (LM) and brain metastasis (BM) of breast cancer. k) The potential biological functions and relevant signaling pathways of seven liver metastasis cancer cell subclusters were evaluated by GSVA based on hallmark gene sets. l) The potential biological functions and relevant signaling pathways of five brain metastasis cancer cell subclusters were evaluated by GSVA based on hallmark gene sets.

Next, we explored the heterogeneity of cancer cells by analyzing the transcriptome patterns and clustering subpopulations. The InferCNV method was used to distinguish neoplastic cells from normal epithelial cells. A total of 24187 malignant epithelial cells in breast cancer liver and brain metastatic tumors were identified. Thirteen main cancer cell subclusters were identified and annotated based on differentially expressed genes (**Figure** **2**a). Feature plots and heatmaps were generated to visualize the most variable genes of each cluster of cancer cells (Figure 2b,c). To investigate the common expression patterns among cancer cells from multiple samples, we established a meta‐cluster algorithm. First, we generated a total of 50 intratumoral expression‐based subclusters after clustering the neoplastic epithelial cells. Next, a hierarchical clustering algorithm was used, and the 50 modules were aggregated into multiple recurrent expression programs. We selected the most activated cancer hallmarks of each program by comparing program cells with nonprogram cells. Six common expression programs with different cell statuses and biological functions were identified, including proliferation‐sustaining, metastasis activation, immune evasion, stress resistance, metabolic reprogramming, and inflammation promotion (Figure 2d,e). The proliferation‐sustaining program (Proliferation) was characterized by high expression of genes associated with the cell cycle (e.g., *MKI67*, *TOP2A*, *CDK1*, *CCNB1*, *KIF4A*, and *E2F1*). The metastasis activation program (Metastasis) consisted of survival‐related genes (*KLF5* and *PDGFRB*), epithelial–mesenchymal transition genes (e.g., *ZEB1* and *YBX1*), and matrix invasion genes (e.g., *MMP2*). The immune evasion program (Immune) was characterized by a series of immune checkpoint genes (e.g., *CD274* and *LGALS3*) and adaptive antitumor immune response genes (e.g., *HLA‐DRA/B1/B5* and *CD74*). The stress resistance program (Stress) consisted of several genes involved in the activation of cell survival signaling pathways (e.g., *JUN*, *IGF1R*, *ERBB2/3*, *PVT1*, and *HIPK2*). The metabolic reprogramming program (Metabolism) had overexpression of genes related to glycolysis, fatty acid processes, and glutathione homeostasis (e.g., *LDHA*, *PFKP*, *PKM*, *PGK1*, *FABP5*, *HSPB1*, and *SLC3A2*). The inflammation promotion program (Inflammation) included genes encoding chemokines (e.g., *CCL3/5/18* and *CXCL1/8*) and interleukins (*IL‐16*). We hierarchically clustered the samples according to the score of each program (Figure 2f). The distribution of each expression program is displayed in a reduced‐dimension map (Figure 2g). Additionally, we quantified the program scores based on the proportion of corresponding program cells to evaluate the activity of these programs in each sample (Figure 2h). Dependency analysis was conducted to validate the pairwise interactions among the expression programs and identified three significant cooccurring program pairs as well as nine reciprocally exclusive program pairs with odds ratios >0.1 and <−0.1, respectively (Figure 2i). We further conducted bioinformatic analysis and biological experiments to investigate whether the top expressed program gene *KLF5* could be a potential target for inhibiting breast cancer metastasis. The TCGA database showed that *KLF5* mRNA was overexpressed in breast cancer, especially in HER2‐positive and triple‐negative breast cancer, the two subtypes with the highest probability of metastasis (Figure S3a, Supporting Information). High expression of *KLF5* was associated with worse distant metastasis‐free survival and overall survival of breast cancer patients in public database cohorts (Figure S3b, Supporting Information). ML264 is a small molecule KLF5 inhibitor with good biological activity and has excellent clinical practice potential.^[^ ^21^ ^]^ We next validated the efficacy of ML264 in suppressing breast cancer metastasis. As revealed by transwell migration assay, the metastatic ability of MDA‐MB‐231 and MCF‐7 breast cancer cells was greatly decreased after being treated with KLF5 inhibitor ML264 (Figure S3c,d, Supporting Information). Wound healing experiment showed that ML264 significantly attenuated the migration ability of breast cancer cells (Figure S3e,f, Supporting Information). To evaluate the effect of ML264 on breast cancer cell colonization ability, we performed colony formation assay. Our data revealed that breast cancer cells' colonization ability was remarkably reduced after being treated with ML264 (Figure S3g,h, Supporting Information). These results suggest that KLF5 inhibitor may become an effective drug to inhibit breast cancer metastasis in the future. To further resolve the heterogeneity of cancer cells in liver‐ or brain‐metastatic breast cancer tissues, we performed an unsupervised clustering analysis for each type of sample. Seven and five cancer cell subclusters were identified and annotated based on differentially expressed genes in liver and brain metastases, respectively (Figure 2j). GSVA pathway enrichment analysis was carried out to explore the potential biological functions and relevant signaling pathways of each cell type using hallmark pathway sets. LM_c2_CD24 showed enrichment of IFN‐*γ* signaling pathways and angiogenesis; LM_c5_CD24 showed enrichment of the WNT beta‐catenin signaling pathway; LM_c6_HMGB2 and LM_c7_MKI67 showed enrichment of the G2M cell cycle checkpoint (Figure 2k). BM_c1_NME2 was enriched with IFN‐*α* and IFN‐*γ* signaling pathways; BM_c4_PROM1 showed heightened activities of Hedgehog, Notch, and JAK‐STAT3 signaling pathways (Figure 2l).

### 2.3 Copy Number Variation and Clonal Evolution Analysis of Metastatic Breast Cancer Cells

**Figure 3:**
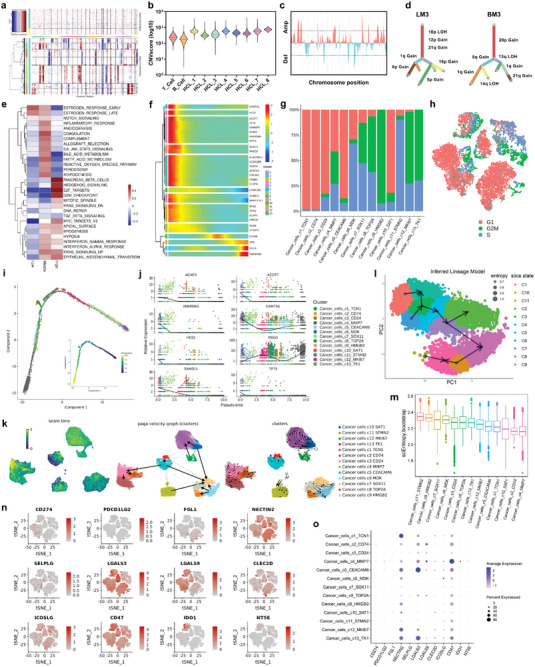
Copy number variation (CNV) and clonal evolution analysis of metastatic breast cancer cells. a) Hierarchical heatmap showing large‐scale CNVs of cancer cells and T/B cells from liver and brain metastases of breast cancer. T/B cells were included as a control reference. Red: gains; blue: losses. b) Eight clusters of cancer cells (HCL1‐8) were identified with similar copy number alterations. The log‐transformed CNV score was used to compare the CNVs of each cluster to those of T cells and B cells. c) CNVs were inferred according to the spanning position of each chromosome (*x*‐axis). d) Clonality trees of the single cancer cells. The branches are delineated according to the percentage of cells in the subclone containing the corresponding CNVs. e) GSVA was performed to compare the differences in pathways in cells with low, median, and high CNV scores. The Z score was used to normalize each column. f) The differentially expressed genes and pseudotime curve are shown in a hierarchical heatmap. g) The percentage of cancer cells in the G1, G2M, and S phases in each cell cluster. The G1 phase was defined as noncycling, and the G2M and S phases were defined as cycling states. h) t‐SNE plot showing the cancer cells in the G1, G2M, and S phases. i) Monocle pseudotime trajectory analysis of cancer cells with highly variable genes. Each dot on the pseudotime curve represents one single cell and is colored according to its cluster label. j) The expression of the most variable genes involved in the cancer cell state transition is shown. k) RNA velocity analysis was performed to investigate the developmental lineages and cellular dynamics of cancer cells. l,m) The entropy of gene expression based on single‐cell RNA expression profiles was explored by the SLICE algorithm to assess and score the stemness of each cancer cell cluster. n) Feature plots showing the normalized expression of immune checkpoint genes in each cancer cell. o) Dot plot showing the expression level of immune checkpoint genes in each cancer cell subcluster.

Breast cancer is largely driven by changes in gene copy number; therefore, we next investigated scRNA‐seq data to infer copy number alterations in cancer cell populations. The inferred CNV profiles revealed interlesion and intralesion heterogeneity in the liver‐ and brain‐metastatic breast cancer tissues (**Figure** **3**a). We clustered the cancer cells with copy number alterations and compared each cluster to the T cells and B cells according to the CNV score (Figure 3b). Most cancer cells possessed amplifications in chromosomes 16/17 and deletions in chromosomes 11/13 (Figure 3c). Multiple canonical (with a CNV percent more than 90%) and noncanonical (with a CNV percent less than 90%) CNVs in subclones were identified in each of the patients, and these CNVs were found in the subclones of cellular populations in cancer cell evolution (Figure 3d). GSVA revealed that pathways related to the G2M cell cycle checkpoint, mitotic spindle, E2F target, epithelial–mesenchymal transition, and Hedgehog signaling were enriched in the CNV‐high group, while pathways related to DNA repair and ROS neutralization were significantly downregulated (Figure 3e). The cell cycle phase scores were calculated based on canonical markers. The results revealed that the cancer cell subclusters c8_TOP2A, c9_HMGB2, c12_MKI67, and c13_TK1 were dominantly in a cycling state, while c1_TCN1, c2_CD74, c3_CD24, c5_CEACAM6, and c10_SAT1 were mainly in a quiescent state (Figure 3g,h). To infer the cancer cell maturation course, we used the Monocle 2 method to perform trajectory analysis of the cancer cells (Figure 3i). In addition, the changes in gene patterns involved in cancer cell state transitions were dissected (Figure 3f,j). To further analyze the time‐resolved phenomena of tumor cell evolution, the RNA velocity method was employed to investigate developmental lineages and cellular dynamics. Notably, four subpopulations of cancer cells (c4, c5, c6, and c12) presented developing trends toward four other clusters of cancer cells (c1, c2, c3, and c7) (Figure 3k). To assess and score the stemness of each cancer cell cluster, we explored the entropy of gene expression based on single‐cell RNA expression profiles by the SLICE algorithm (Figure 3l). We found that the cancer cell subpopulation c11_STMN2 had the highest entropy score, while the cancer cell subpopulation c4_MMP7 had the lowest entropy score (Figure 3m). Furthermore, we explored the expression of immune checkpoint ligands in each cancer cell subpopulation. The most promising immune checkpoint therapeutic targets, CD274 (PD‐L1) and PDCD1LG2 (PD‐L2), were expressed at low levels in each cancer cell population (Figure 3n,o). Two other immune checkpoint ligands, *NECTIN2* and *LGALS3*, were highly expressed in almost all subclusters. Interestingly, as an antiphagocytic molecule, *CD47* also exhibited very high expression in cancer cells (Figure 3n,o). These surface proteins are expected to be immune target for future liver‐ and brain‐metastatic breast cancer treatments.

### 2.4 The Landscape of Lymphocytes and Innate Lymphoid Cells in Breast Cancer Liver and Brain Metastases

**Figure 4:**
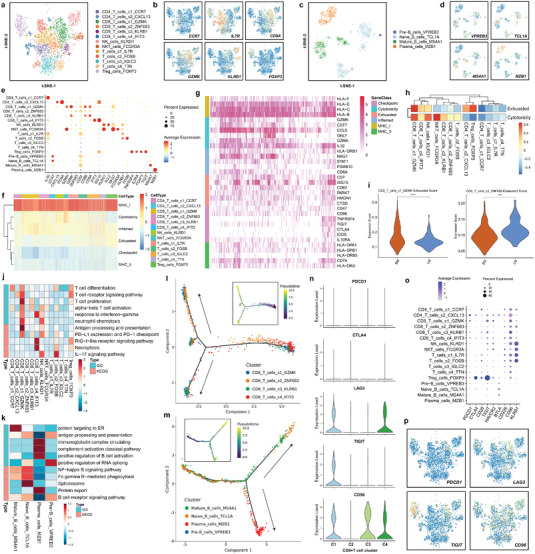
Landscape of lymphocytes and innate lymphoid cells in breast cancer liver and brain metastases. a) t‐SNE plot of the T cell and NK cell landscape colored by subcluster. b) Feature plots showing the normalized expression of canonical marker genes in each T cell and NK cell subcluster. c) t‐SNE plot of the B cell landscape colored by subcluster. d) Feature plots showing the normalized expression of canonical marker genes in each B cell subcluster. e) Dot plot showing the expression level of canonical marker genes across all lymphocyte and innate lymphoid cell subtypes. f) Heatmap showing the gene set score of each T cell and NK cell according to GSVA. Gene sets included MHC molecules, T cell cytotoxicity, T cell exhaustion, inflammatory pathway, regulatory cytokines, and immune checkpoint pathway sets. g) Heatmap showing the expression of core genes in each gene set. h) The cytotoxicity and dysfunction of each T cell and NK cell cluster were analyzed and quantified based on gene signature scores. i) Exhaustion scores of CD8_T_cells_c1_GZMK and CD8_T_cells_c2_ZNF683 in liver and brain metastases of breast cancer. j,k) The potential biological functions and relevant signaling pathways of T, B, and NK cell subclusters were evaluated by GO and KEGG analyses. l) Monocle pseudotime trajectory analysis of CD8+ T cells (CD8_T_cells_c1_GZMK, CD8_T_cells_c2_ZNF683, CD8_T_cells_c3_KLRB1, and CD8_T_cells_c4_IFIT3) with highly variable gene expression. l) Monocle pseudotime trajectory analysis of B cells (Pre‐B_cells_VPREB3, Naive_B_cells_TCL1A, Mature_B_cells_MS4A1, and Plasma_cells_MZB1) with highly variable gene expression. o) Violin plot showing the expression level of immune checkpoint genes in four CD8+ T cell subclusters. o) Dot plot showing the expression level of immune checkpoint genes in each lymphocyte and innate lymphoid cell subcluster. p) Feature plots showing the normalized expression of immune checkpoint genes in lymphocytes and innate lymphoid cells.

To explore the immune milieu of breast cancer liver and brain metastases, we separately reclustered T cells and B cells to identify subtypes at high resolution. Thirteen T cell and innate lymphoid subclusters were identified across all patients (**Figure** **4**a,b and Figure S4a, Supporting Information). We found that liver‐metastatic lesions had higher infiltration of NK cells and CD8+ T cells than brain‐metastatic lesions (Figure S4b, Supporting Information). CD4+ T cell clusters consisted of regulatory T cells (Treg_cells_FOXP3), naive/central memory T cells (CD4_T_cells_c1_CCR7), and follicular helper T cells (CD4_T_cells_c2_CXCL13). Among the four CD8+ T cell clusters, two had high expression of immune checkpoint molecules, including *TIGIT*, *LAG3*, and *CD96* (CD8_T_cells_c1_GZMK and CD8_T_cells_c4_IFIT3). Additionally, natural killer (NK) cells (NK_cells_KLRD1) and natural killer T (NKT)‐like cells (NKT_cells_FCGR3A) were also characterized by the expression of NK markers and *αβ* T cell receptor. After the B cell reclustering, four major subpopulations were identified across all patients, including Pre‐B_cells_VPREB3, Naive_B_cells_TCL1A, Mature_B_cells_MS4A1, and Plasma_cells_MZB1 (Figure 4c,d). Brain metastases had higher infiltration of naive B cells than liver metastases of breast cancer, while the abundance of plasma cells in liver metastases of breast cancer was higher than that in brain metastases (Figure S4b, Supporting Information). The expression of canonical markers in each immune cell subtype is summarized in a dot plot (Figure 4e). CD4+ T cells exhibited relatively high expression levels of costimulatory molecules, including *ICOS* and *TNFRSF4*, which are important for the cytotoxic stimulation of CD8+ T cells (Figure 4f,g). CD8+ T cells had a high expression level of several cytotoxicity genes, including *GZMB*, *GZMK*, and *GZMH*. Notably, the data also revealed that these subtypes of CD8+ T cells positively expressed T cell exhaustion‐related inhibitory receptors, including *LAG3*, *TIGIT*, and *CD96*, indicating that these CD8+ T cells were exhausted after the initial activation step in the liver and brain metastases of breast cancer (Figure 4f,g). The CD8_T_cells_c1_GZMK and CD8_T_cells_c4_IFIT3 clusters possessed substantially higher dysfunction scores than the other two clusters (CD8_T_cells_c2_ZNF683 and CD8_T_cells_c3_KLRB1) (Figure 4h and Figure S4c, Supporting Information). Moreover, the CD8_T_cells_c1_GZMK cluster exhibited higher dysfunction scores in brain metastases than in liver metastases (Figure 4i). Gene Ontology (GO) and Kyoto Encyclopedia of Genes and Genomes (KEGG) analyses were conducted to explore the potential biological functions and relevant signaling pathways of each cell type. The CD8_T_cells_c1_GZMK cluster was enriched in T cell proliferation, response to interferon‐gamma, neutrophil chemotaxis, and immune checkpoint pathways, while the CD8_T_cells_c4_IFIT3 cluster was associated with necroptosis, response to interferon‐gamma, and the RIG‐I‐like receptor signaling pathway (Figure 4j). Naive_B_cells_TCL1A showed heightened activities of NF‐kappa B signaling pathway, the spliceosome, and Fc gamma R‐mediated phagocytosis, while the Plasma_cells_MZB1 cluster showed enrichment of pathways related to immunoglobulin complex circulation, classical complement activation, B cell activation, and protein export (Figure 4k). To trace the evolution of CD8+ T cells, pseudotime trajectory analysis was conducted. The CD8_T_cells_c1_GZMK cluster had the most terminal status and highest pseudotime score, and two developmental trajectories of the CD8_T_cells_c2_ZNF683 and CD8_T_cells_c3_KLRB1 clusters were revealed (Figure 4l and Figure S4d, Supporting Information). For the pseudotime trajectory analysis of B cells, the Plasma_cells_MZB1 cluster was in the most terminal phase and had the highest pseudotime score (Figure 4l and Figure S4e, Supporting Information). We next assessed the expression of immune checkpoint receptors in each CD8+ T cell subpopulation and found that the expression of *LAG3*, *TIGIT*, *CD96*, and *KLRB1* (*CD161*) in CD8+ T cells was higher than that of *PDCD1* (*PD‐1*) (Figure 4n–p). Cell–cell signaling interactions between cancer cells and immune cells were predicted based on known ligand–receptor pairs by CellphoneDB. Treg cells had the most interactions with other cell populations, showing robust interactions with cancer cells (Figure S4f, Supporting Information). Cancer cells recruited the CD4_T_cells_c2_CXCL13, CD8_T_cells_c1_GZMK, CD8_T_cells_c4_IFIT3, NK_cells_KLRD1, and Treg_cells_FOXP3 clusters through the CXCL10‐CXCR3 ligand–receptor pair (Figure S4g, Supporting Information). Other significant ligand–receptor pairs involved in cell–cell communications are displayed in Figure S4h (Supporting Information). To verify our results in a larger sample size, we extracted bulk transcriptome data and corresponding clinical information from the Gene Expression Omnibus (GEO) database (ID: GSE56493, GSE12276, GSE46141, and GSE173661). A total of 421 cases of distant metastatic breast cancer were analyzed, including 61 cases of brain metastasis and 43 cases of liver metastasis. We used the “xCell” algorithm to calculate the tumor microenvironment components based on bulk transcriptome data (Figure S5a, Supporting Information). We found that liver metastasis had higher infiltration of NK cells, CD4+ effective T cells, and CD8+ effective T cells than brain metastasis (Figure S5b, Supporting Information). The correlation between each immune checkpoint gene and tumor microenvironment cell was also calculated (Figures S6a and S7b, Supporting Information). The expression of classical immune checkpoint genes *CTLA‐4*, *CD274* (*PD‐L1*), *PDCD1LG2* (*PD‐L2*), and *PDCD1* (*PD‐1*) were lower in brain/liver metastasis than that in other metastatic sites (Figure S6b, Supporting Information). Other immune checkpoint ligands or receptors, *SELPLG*, *HAVCR2*, *LGALS3*, and *LGALS9*, were highly expressed in cancer cells or other infiltrating cells (Figure S6b, Supporting Information). These bulk transcriptome data from large samples further confirmed the results of our single‐cell RNA sequencing.

### 2.5 Characterization of Immunosuppressive Myeloid Cells in the Tumor Microenvironment

**Figure 5:**
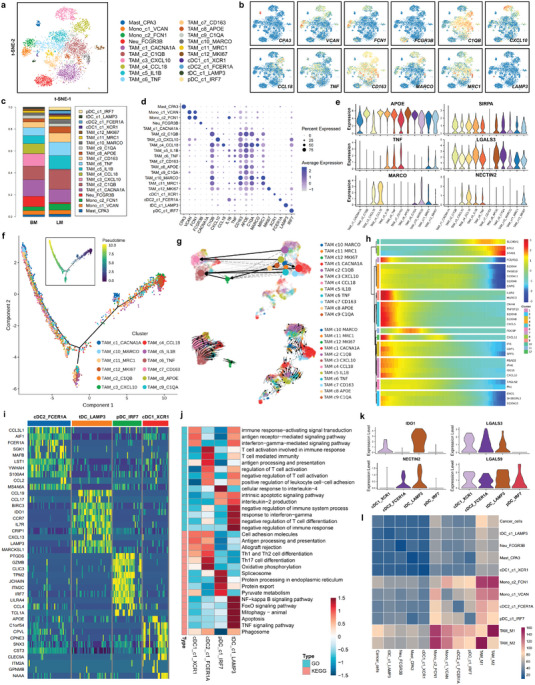
Characterization of immunosuppressive myeloid cells in the tumor microenvironment. a) Reclustering of myeloid cells and visualization of the profile of each cell subtype via a t‐SNE plot. b) Feature plots showing the normalized expression of canonical marker genes in each myeloid cell subcluster. c) The relative proportions of each myeloid cell cluster in liver and brain metastases of breast cancer. d) Dot plot showing the expression level of canonical marker genes across all myeloid cell subtypes. e) Violin plot showing the expression levels of a macrophage canonical marker (*APOE*), an M1 macrophage marker (*TNF*), an M2 macrophage marker (*MARCO*), a phagocytic inhibitory molecule (*SIRPA*), and immune checkpoint genes (*LGALS3* and *NECTIN2*) in twelve tumor‐associated macrophage (TAM) subclusters. f) Monocle pseudotime trajectory analysis of tumor‐associated macrophages (TAMs) with highly variable gene expression. Each dot on the pseudotime curve represents one single cell and is colored according to its cluster label. g) RNA velocity analysis was performed to investigate the developmental lineages and cellular dynamics of TAMs. h) The differentially expressed genes and the TAM pseudotime curve are shown in a hierarchical heatmap. i) Heatmap of the expression levels of the top 10 differentially expressed genes among the four subclusters of dendritic cells (DCs). j) The potential biological functions and relevant signaling pathways of each DC subcluster were evaluated by GO and KEGG analyses. k) Violin plot showing the expression levels of an immune suppression gene (*IDO1*) and immune checkpoint genes (*LGALS3*, *LGALS9*, and *NECTIN2*) in four DC subclusters. l) Heatmap showing the number of cell–cell interactions between myeloid cells and cancer cells, predicted by the CellphoneDB 2 method.

Myeloid cells consisted of monocytes, mast cells, neutrophils, tumor‐associated macrophages (TAMs) and dendritic cells (DCs), and these cells could be divided into 20 clusters (**Figure** **5**a). The markers and proportion of each myeloid cell subtype are shown in feature plots and histograms (Figure 5b,c). The scaled relative expression levels and proportion of the cluster‐specific markers in each cell subpopulation are displayed in dot plots (Figure 5d). Monocytes formed two clusters: Mono_c1_VCAN and Mono_c2_FCN1. Bulk transcriptome data indicated that the abundance of monocytes was higher in liver metastasis than that in other metastatic sites (Figure S5b, Supporting Information). TAMs formed 12 clusters that could be divided into M1 macrophages (TAM_c5‐7), M2 macrophages (TAM_c1‐4 and TAM_c8‐11), and proliferating macrophages (TAM_c12). M1‐TAMs are associated with inflammatory factors, including *CCL2/3/4*, *CXCL3*, and *TNF*, which recruit T cells, immature DCs, and NK cells in the tumor microenvironment. M2‐TAMs are tumor‐promoting macrophages and showed high expression of *MRC1*, *CD163*, *MARCO*, and *MAF* (Figure 5e). In addition, M2‐like TAMs had higher expression of immune checkpoint molecules, including *PD‐L1*, *PD‐L2*, *LGALS3*, *LGALS9*, *NECTIN2*, *SELPLG*, *CLEC2D*, and *TNFRSF14*, than M1‐like TAMs (Figure 5e and Figure S8a,b, Supporting Information). The antiphagocytic receptor coding‐gene *SIRPA* exhibited a higher expression level in M2‐like TAMs than in M1‐like TAMs (Figure 5e and Figure S8a,b, Supporting Information). An immunosuppressive subcluster of TAMs, a CCL18+ M2‐like macrophage subcluster, was also identified, which expressed a high level of several negative immune regulators (Figure S4b, Supporting Information). Bulk transcriptome data indicated that the abundance of M2‐like TAMs was higher in brain and liver metastasis than in other metastatic sites (Figure S5b, Supporting Information). Similarly, *SIRPA* and *CD47* expression were higher in brain and liver metastases than in other metastases (Figure S6b, Supporting Information). M2‐like TAM level was negatively correlated with CD8+ effective T cell and NK cell levels in breast cancer metastasis (Figure S7a, Supporting Information). The Monocle 2 program was used to analyze the trajectory of TAM maturation (Figure 5f), and the changes in gene expression patterns involved in TAM state transitions were identified (Figure 5h and Figure S8c, Supporting Information). The RNA velocity algorithm was further used to investigate the TAM developmental lineages and cellular dynamics according to the time‐resolved phenomena of TAM evolution (Figure 5g and Figure S8d, Supporting Information). DCs consisted of four clusters: conventional DCs (cDC1_c1_XCR1 and cDC2_c1_FCER1A), tolerogenic DCs (tDC_c1_LAMP3) and plasmacytoid DCs (pDC_c1_IRF7). cDCs are mature DCs that may activate CD8+ T cells and other antitumor immune responses, and they had high expression of costimulatory factors, MHC class II molecules, and proinflammatory cytokines (Figure 5i). pDCs level positively associated with CD8+ effective T cell and NK cell levels in breast cancer metastasis (Figure S7a, Supporting Information). LAMP3‐positive tDCs are an immunosuppressive subcluster of DCs, and they expressed high levels of several negative immune regulators, including *IDO1*, *CCR7*, *LGALS3*, *LGALS9*, and *NECTIN2* (Figure 5i,k). GO and KEGG analyses were performed to explore the potential biological functions and relevant signaling pathways of each cell type. The cDC1_c1_XCR1 and cDC2_c1_FCER1A clusters were enriched in T cell activation, antigen processing and presentation, and regulation of leukocyte adhesion, while the tDC_c1_LAMP3 cluster was associated with negative regulation of the immune response and T cell differentiation (Figure 5j). Pseudotime trajectory analysis revealed that tDC_c1_LAMP3 and pDC_c1_IRF7 were derived from cDC1_c1_XCR1 and cDC2_c1_FCER1A (Figure S8e–g, Supporting Information). According to the CellphoneDB analysis, TAMs had the most interactions with cancer cells and other myeloid cells in the tumor microenvironment (Figure 5l).

### 2.6 Diversity of Cancer‐Associated Fibroblasts (CAFs), Endothelial Cells (ECs), and Mural cells (MCs) in the Tumor Microenvironment

**Figure 6:**
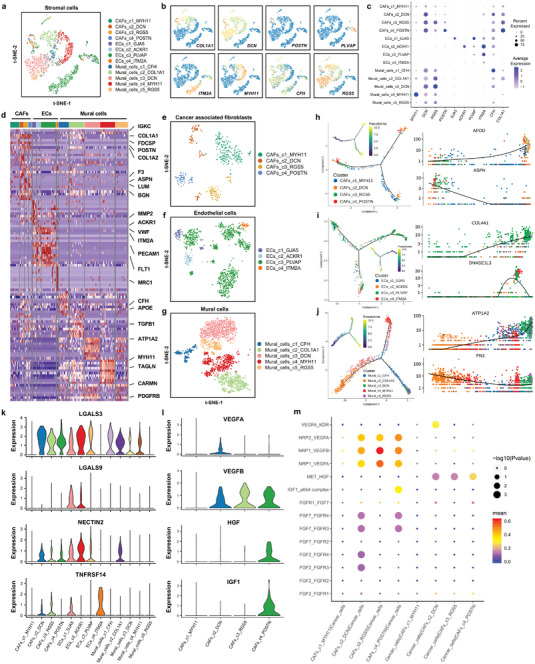
Diversity of cancer‐associated fibroblasts (CAFs), endothelial cells (ECs) and mural cells (MCs) in the tumor microenvironment. a) t‐SNE plot of the stromal cell landscape colored by subcluster. b) Feature plots showing the normalized expression of canonical marker genes in each stromal cell subcluster. c) Dot plot showing the expression level of canonical marker genes across all stromal cell subtypes. d) Heatmap of the expression levels of the top 10 differentially expressed genes among the four subclusters of stromal cells. e) Reclustering of cancer‐associated fibroblasts (CAFs) and visualization of the profile of each subtype via a t‐SNE plot. f) Monocle pseudotime trajectory analysis of CAFs with highly variable gene expression. Each dot on the pseudotime curve represents one single cell and is colored according to its cluster label. g) Reclustering of endothelial cells (ECs) and visualization of the profile of each subtype via a t‐SNE plot. h) Monocle pseudotime trajectory analysis of ECs with highly variable gene expression. Each dot on the pseudotime curve represents one single cell and is colored according to its cluster label. i) Reclustering of mural cells (MCs) and visualization of the profile of each subtype via a t‐SNE plot. j) Monocle pseudotime trajectory analysis of MCs with highly variable gene expression. Each dot on the pseudotime curve represents one single cell and is colored according to its cluster label. k) Violin plot showing the expression levels of immune checkpoint genes (*LGALS3*, *LGALS9*, *NECTIN2*, and *TNFRSF14*) in each stromal cell subtype. l) Violin plot showing the expression levels of cell growth factor genes (*VEGFA*, *VEGFB*, *HGF*, and *IGF1*) in stromal cell subtypes. m) Dot plot showing the ligand–receptor pairs of cell growth factors between cancer cells and each CAF cluster predicted by the CellphoneDB 2 method.

Three major cell types were identified in the stromal compartment: CAFs (gene markers: *COL1A1*, *COL1A2*, *POSTN*, and *PDGFRA*), ECs (gene markers: *GJA5*, *ACKR1*, *PLVAP*, and *ITM2A*), and MCs (gene markers: *MYH11* and *PDGFRB*) (**Figure** **6**a–d and Figure S9a, Supporting Information). According to the most variable genes, CAFs were clustered into four subpopulations (CAFs_c1_MYH11, CAFs_c2_DCN, CAFs_c3_RGS5, and CAFs_c4_POSTN) (Figure 6e and Figure S9b, Supporting Information). ECs were clustered into four subpopulations: arterial endothelial cells (ECs_c1_GJA5), venous endothelial cells (ECs_c2_ACKR1), capillary endothelial cells (ECs_c3_PLVAP), and liver sinusoidal endothelial cells (ECs_c4_ITM2A) (Figure 6f and Figure S9c, Supporting Information). MCs were clustered into five subpopulations: Mural_cells_c1_CFH, Mural_cells_c2_COL1A1, Mural_cells_c3_DCN, Mural_cells_c4_MYH11, and Mural_cells_c5_RGS5 (Figure 6g and Figure S9d, Supporting Information). Principal component analysis and diffusion map analysis revealed two distinct evolutionary trajectories of CAFs_c2_DCN, which ultimately developed into an inflammatory‐like cluster of CAFs (CAFs_c1_MYH11) (Figure 6h). Arterial endothelial cells (ECs_c1_GJA5), venous endothelial cells (ECs_c2_ACKR1), and liver sinusoidal endothelial cells (ECs_c4_ITM2A) developed from capillary endothelial cells (ECs_c3_PLVAP), which are involved in the dynamic angiogenesis process in metastatic lesions (Figure 6i). Mural_cells_c2_COL1A1 cells developed into two branches, and the Mural_cells_c4_MYH11 and Mural_cells_c5_RGS5 clusters within these branches had the highest pseudotime scores, representing the most differentiated and matured MCs (Figure 6j). RNA velocity analysis also confirmed the transition of each cell type based on the time‐resolved phenomena (Figure S9e, Supporting Information). Next, we examined the expression of immune checkpoint ligands in each cancer cell subpopulation. Several immune checkpoint molecules were highly expressed in the three stromal cell subtypes, including *LGALS3*, *LGALS9*, *NECTIN2*, and *TNFRSF14* (Figure 6k and Figure S9f, Supporting Information). According to the CellphoneDB analysis, CAFs_c2_DCN, ECs_c1_GJA5, and Mural_cells_c2_COL1A1 were the clusters of CAFs, ECs, and MCs, respectively, with the most interactions with cancer cells (Figure S9g, Supporting Information). Moreover, we found that different CAF subpopulations had different growth factor secretion patterns (Figure 5l). The data suggested that CAFs could produce VEGFRA/B, which binds to VEGF receptors on ECs to promote angiogenesis. Based on the cell growth factor and receptor pairs, we constructed a regulatory network for CAFs and cancer cells. The CAFs_c2_DCN subcluster promoted cancer cell growth via the FGF2/7‐FGFR3/4 and IGF1‐IGF1R signaling pathways (Figure 5m). GO and KEGG analyses were performed to explore the potential biological functions and relevant signaling pathways of each stromal cell type (Figure S9j–l, Supporting Information). In particular, the RGS5+ cancer‐associated fibroblast cluster was identified and has been proven to negatively regulate the immune process in metastatic breast cancer by secreting periostin and biglycan.^[^ ^22^ ^]^ We also confirmed that the level of CAF was negatively correlated with CD8+ effective T cell and NK cell level via bulk transcriptome analysis (Figure S7a, Supporting Information).

### 2.7 Cell Clustering and Functional Annotation of Organ‐Specific Resident Cells in Breast Cancer Liver and Brain Metastases

Resident cells (microglial cells, astrocytes, oligodendrocytes, neurons, hepatocytes, etc.) are indispensable components of the brain or liver metastasis microenvironment that participate in forming the unique colonized niche and immune milieu.^[^ ^23^, ^24^, ^25^, ^26^ ^]^ We identified thirteen cell clusters, including microglial cells (Microglials_c1_CCL4 and Microglials_c2_LGALS1), astrocytes (Astrocytes_c1_SLC4A4), oligodendrocytes (c1_KLK6, c2_CNP, c3_TMEM63A, c4_KIF6, and c5_OLIG2), neurons (Neurons_c1_SYT1), oligodendrocyte progenitor cells (OPCs_c1_PTPRZ1), hepatocytes (Hepatocytes_c1_TGFB1 and Hepatocytes_c2_ALB), and cholangiocytes (Cholangiocytes_CFTR) (Figure S10a, Supporting Information). The markers and proportion of each cell subtype are shown in feature plots, histograms, bubble plots and heatmaps (Figure S10b–e, Supporting Information). Microglials_c1_CCL4 promoted the immune response by secreting chemokines such as CCL3/4 to recruit T cells, immature DCs, and NK cells in the tumor microenvironment. Microglials_c2_LGALS1 expressed a high level of galectin‐1, which likely fosters an immunosuppressive microenvironment via CD8+ T cell exclusion.^[^ ^27^, ^28^ ^]^ In addition, we found that the immune checkpoint genes *SELPLG* and *LGALS3* were highly expressed in microglial cells, while FGL1, *NECTIN2*, and *LGALS3* were highly expressed in hepatocytes and cholangiocytes (Figure S10f,g,h, Supporting Information). Next, we performed GO and KEGG analyses to explore the potential biological functions and signaling pathways of each cell cluster. The Microglials_c1_CCL4 cluster was enriched in the chemokine signaling pathway and neutrophil activation involved the immune response, while the Microglials_c2_LGALS1 cluster was associated with negative regulation of T cell activation and immune checkpoint pathways (Figure S10i, Supporting Information). Pseudotime trajectory analysis revealed that Oligodendrocytes_c1_KLK6, Oligodendrocytes_c2_CNP, and Oligodendrocytes_c5_OLIG2 were derived from Oligodendrocytes_c3_TMEM63A and Oligodendrocytes_c4_KIF6 (Figure S11a,b, Supporting Information). Dynamic changes in gene expression with pseudotime variation were identified via heatmap and trajectory chart analyses (Figure S11c,d, Supporting Information). The RNA velocity analysis indicated that Oligodendrocytes_c3_TMEM63A and Oligodendrocytes_c4_KIF6 are likely upstream of other effectors; however, bidirectional transitions between each cluster could also occur (Figure S11e,f, Supporting Information). Cell interactions between cancer cells and resident cells in different metastatic sites were predicted based on known ligand–receptor pairs by CellphoneDB analysis (Figure S12a–c, Supporting Information). Microglial cells had the most interactions with cancer cells, while neurons had the fewest interactions with other cell populations (Figure S12d, Supporting Information). Due to the important role of cell growth factor in the tumor microenvironment, we further analyzed its expression. *VEGFA/B*, *IGF1*, *HGF*, and *FGF20* were the most highly expressed cell growth factors secreted by microglial cells (Figure S12e, Supporting Information). These results uncovered the roles of resident cells in promoting cancer cell colonization and forming an immunosuppressive microenvironment in liver and brain metastases of breast cancer.

## 3 Discussion

Due to the clonal evolution of cancer cells, immune‐phenotype shifts of infiltrating cells, and organ‐specific niche, metastatic tumors exhibit a more profound immunosuppressive microenvironment than primary tumors.^[^ ^29^, ^30^ ^]^ Tumor cells can encourage normal resident cells to become tumor‐promoting cells, which facilitate the colonization, growth, and immune evasion of cancer cells.^[^ ^31^ ^]^ Increasing the understanding of metastatic tumors and their ecosystem might contribute to the development of more precise treatment and provide cancer immunotherapy benefits to more patients.^[^ ^32^ ^]^ With the advances made in high‐throughput single‐cell sequencing technology and bioinformatic analysis, scientists can depict the landscape of and investigate the cell– communications in the tumor microenvironment at a single‐cell resolution.^[^ ^33^, ^34^, ^35^ ^]^ With single‐cell sequencing, the unique architecture and cellular composition of the tumor ecosystem in primary breast cancer have been widely studied, which is crucial for guiding immune‐phenotype classification and uncovering the mechanisms of primary breast cancer resistance to immunotherapy.^[^ ^17^, ^18^ ^]^ However, the key determinants and biological mechanisms underpinning the distant metastatic human breast cancer immune microenvironment remain elusive.

In the present study, we first provided a high‐resolution analysis of liver‐ and brain‐metastatic breast cancer samples, which identified diverse major cell types, including cancer cells, lymphocytes, myeloid cells, stromal cells, and organ‐specific resident cells. The molecular features, regulators, dynamics, and functions of each cell cluster were analyzed regarding their role in the progression and immune escape of metastatic tumors. Six common expression programs with different cell statuses and biological functions were identified in cancer cells, including proliferation sustaining, metastasis activation, immune evasion, stress resistance, metabolic reprogramming, and inflammation promotion. Among these metastasis‐related hub genes, *KLF5* was identified and validated as an effective target for inhibiting breast cancer metastasis. KLF5 is a zinc‐finger transcription factor highly expressed in several malignancies, which has recently become a popular target in cancer research.^[^ ^36^, ^37^, ^38^, ^39^ ^]^ We also found that KLF5 small molecule inhibitor ML264 can effectively inhibit breast cancer metastasis, and it is expected to be used in the clinic in the future. Furthermore, several immunosuppressive cells were identified to reprogram the metastatic ecosystem, including FOXP3+ regulatory T (Treg) cells, LAMP3+ tolerogenic DCs (tDCs), CCL18+ M2‐like macrophages, RGS5+ cancer‐associated fibroblasts, and LGALS1+ microglial cells. Treg cells are a subset of T cells with significant immunosuppressive effects and phenotypic characteristics, such as Foxp3, CD25, and CD4 expression. They inhibit the antitumor effects of the immune response by secreting a variety of inhibitory cytokines (such as TGF‐*β*, IL‐10, and IFN‐*γ*) and blocking costimulatory ligands to suppress subsequent T cell activation.^[^ ^40^ ^]^ We also identified an immunosuppressive subcluster of DCs (LAMP3+ tDCs) that inhibited the immune response by expressing a high level of several negative immune regulators, including *IDO1*, *CCR7*, *LGALS3*, *LGALS9*, and *NECTIN2*. According to previous studies, LAMP3+ tDCs are enriched in tumor tissues and can inhibit the activation of CD8+ T cells by recruiting Treg cells and other CCR4+ immune cells to tumor regions.^[^ ^41^ ^]^ Another immunosuppressive cluster of myeloid cells, CCL18+ M2‐like macrophages, are more likely to exist in the metastatic microenvironment due to hypoxia induction and metabolic reprogramming.^[^ ^42^ ^]^ It is well established that cancer‐associated fibroblasts remodel the extracellular matrix and promote cancer immune evasion in the tumor microenvironment. We found that the RGS5+ cancer‐associated fibroblast cluster could secrete periostin and biglycan, which are negative regulators of the immune process in metastatic breast cancer.^[^ ^22^ ^]^ Interestingly, an LGALS1+ microglial cell subcluster was identified in brain metastases of breast cancer expressing a high level of galectin‐1, which can foster an immune‐exclusive microenvironment by reprogramming Treg cells.^[^ ^27^, ^28^ ^]^ Moreover, metastatic cancer cells have a high expression level of CD47, which interacts with the macrophage inhibitory receptor signal regulatory protein *α* (SIRP*α*) to transmit a “don't eat me” signal and prevent macrophage phagocytosis.^[^ ^43^, ^44^ ^]^ These cell clusters may also be targets for immunotherapy for liver and brain metastasis of breast cancer.

Immune checkpoint therapy has been proven effective against various advanced solid tumors, and its application has quickly gained momentum in antitumor drug research.^[^ ^45^, ^46^, ^47^ ^]^ Recent studies have revealed that some subgroups of metastatic breast cancer may respond well to anti‐PD‐1/L1 immune checkpoint therapy, although breast cancer was once considered an immune‐quiescent tumor.^[^ ^48^ ^]^ Nevertheless, individuals with liver or brain metastasis respond poorly to anti‐PD‐1/L1 immune checkpoint inhibitors compared to those with metastasis at other sites.^[^ ^5^, ^6^, ^7^ ^]^ Our single‐cell transcriptome data revealed low expression levels of the PD‐1 checkpoint molecule and high expression levels of the *LAG3* and *TIGIT* checkpoint molecules in CD8+ T cells. Furthermore, the immune checkpoint ligands *PD‐L1/2* was barely expressed in cancer cells, immune cells, or stromal cells. The ligands of *LAG3* and *TIGIT*, *LGALS3*, and *NECTIN2*, respectively, were highly expressed in the above cells. These results suggested that the interactions of the immune checkpoint molecules LAG3‐LGALS3 and TIGIT‐NECTIN2 between CD8+ T cells and cancer/immune/stromal cells played a dominant role in the immune escape of liver‐ and brain‐metastatic breast cancer. Galectin‐3 (encoded by *LGALS3*) is produced by both tumor cells and macrophages and can block T cell activation signaling, induce T cell apoptosis, and inhibit IFN‐*γ* secretion by interacting with LAG3 on the cellular surface of T cells.^[^ ^49^ ^]^ CD115 (encoded by *NECTIN2*) binds TIGIT to transduce signals through its intracellular tail immunoreceptor tyrosine‐based inhibitory motif (ITIM) and/or Ig tail‐tyrosine (ITT)‐like motif to inhibit a variety of lymphocytes (effector/regulatory T cells, NK cells, etc.).^[^ ^50^ ^]^ Therefore, targeting these two second‐generation immune checkpoint receptor targets (LAG3 and TIGIT) and their ligands (LGALS3 and NECTIN2) is a potential therapeutic strategy for patients with liver and brain metastasis of breast cancer.

In summary, our study uncovered intratumoral heterogeneity and an immunosuppressive tumor ecosystem in liver‐ and brain‐metastatic breast cancer, which provides insights into the mechanisms underlying immune checkpoint treatment resistance and information for the development of precise immunotherapy strategies for these patients.

## 4 Experimental Section

### Patients and Samples

Liver or brain metastases of breast cancer were collected from six patients undergoing surgery in Sun Yat‐sen University Cancer Center. The clinical information of all included patients with liver or brain metastatic breast cancer are showed in Table S1 (Supporting Information). The pathological and immunohistochemical information of the primary and metastatic tumors are showed in Table S2 (Supporting Information). All samples analyzed in this study were from patients who were pathologically diagnosed with metastatic breast cancer. The specimens for scRNA‐seq were obtained from the tumor area, and the normal liver and brain tissues around the tumor were removed before dissociation. Only female patients with metastatic breast cancer were included. Their ages ranged from 41 to 55 years, with a median age of 50 years. Fresh tumor weights ranged from 150 to 300 mg. None of the patients received chemotherapy or radiation to treat their metastasis before surgery, except for patient BM01 (who achieved pathologic complete response after anti‐HER2 therapy and chemotherapy). This study was approved by the Institutional Research Ethics Committee of Sun Yat‐sen University Cancer Center and conducted under the guidance of the Declaration of Helsinki (G202102201). Informed consent was obtained from all patients.

### Tissue Dissociation and Single‐Cell Suspension Preparation

Fresh tissues were stored in sCelLiveTM Tissue Preservation Solution (Singleron, China) on ice immediately after resection. The tissues were washed with HBSS three times and then digested with 2 mL sCelLiveTM Tissue Dissociation Solution (Singleron) at 37 °C for 15 min. To remove red blood cells, red blood cell lysis buffer (2 mL) was added to the cells and incubated at 25 °C for 10 min. The solution was then centrifuged at 500 × *g* for 5 min and suspended in PBS. Trypan blue (Sigma) staining was used to evaluate cellular viability under a microscope.

### Library Preparation and Single‐Cell RNA Sequencing

Single‐cell suspensions were barcoded using the Chromium Single Cell Library, Gel Bead & Multiplex Kit (10× Genomics). Briefly, cells were partitioned into Gel Beads in Emulsion in the ChromiumTM Controller instrument, where cell lysis and barcoded reverse transcription of RNA occurred. Each DNA library was sequenced on a HiSeq X instrument (Illumina) with 150 bp paired‐end reads.

### Raw Data Processing and Quality Control

After removing low‐quality reads, raw reads were processed to generate gene expression profiles using Cell Ranger v.3.0.2. Reads from the 10× library were mapped to the human genome reference sequence GRCh38 with ensemble version 92 gene annotation. Gene counts and UMI counts were acquired by featureCounts software. Cells were filtered according to the following criteria: gene count below 200, top 2% gene counts, and top 2% UMI counts. Cells with over 50% mitochondrial content were removed. The Seurat program (R package, v.3.0.1)^[^ ^51^ ^]^ was utilized to perform cell type annotation and clustering analysis. A parameter resolution of 1.2 was set for the FindClusters function to conduct clustering analysis.

### Differentially Expressed Gene (DEG) Analysis and Cell Type Annotation

The Seurat v3.1.2 function FindMarkers based on the Wilcoxon likelihood‐ratio test with default parameters was used to identify DEGs. Genes expressed in more than 10% of the cells in a cluster and with an average logFC (fold change) higher than 0.25 were selected as DEGs. According to the SynEcoSys database, the cell type of each cluster was annotated based on the expression of canonical markers found in the DEGs. The Seurat v3.1.2 functions DoHeatmap/Vlnplot/DotPlot were used to visualize the expression of markers identified in each cell type.

### Single‐Cell Copy Number Variation (CNV) Analysis

The InferCNV method^[^ ^52^ ^]^ was used to validate the CNVs and distinguish malignant cells from normal epithelial cells. Nonmalignant cells (T cells and B cells) in our samples were used as control references to evaluate the CNVs of malignant cells. Genes expressed in over 20 cells were sorted according to their position on each chromosome. The relative expression values were centered to 1, using 1.5 standard deviations from the residual‐normalized expression values as the ceiling. A slide window size of 101 genes was used to smooth the relative expression on each chromosome to remove the effect of gene‐specific expression.

### Pathway Enrichment Analysis

Gene Ontology (GO) and Kyoto Encyclopedia of Genes and Genomes (KEGG) analyses were used to explore the potential biological functions and relevant signaling pathways of each cell type. The “clusterProfiler” R package was utilized to conduct GO and KEGG analyses. Only biological process categories were used as a background set in the GO analysis. Pathways or functions with adjusted *P* values < 0.05 were considered significantly enriched items. GSVA was performed using hallmark pathway sets. The average expression of genes in each cell cluster was defined as input data.

### Identification of Expression Programs

The Consensus Non‐negative Matrix factorization (cNMF) algorithm (https://github.com/dylkot/cNMF) was used to extract transcriptional programs by taking the top 100 genes as the meta‐signature and calculating the score of each transcriptional program for each cancer cell based on the meta‐signature. Based on the Pearson correlation coefficient between each program, the meta‐signature was developed, and the samples were hierarchically clustered.

### Trajectory and RNA Velocity Analysis

Pseudotime trajectory analysis was conducted with the Monocle 2 algorithm^[^ ^53^ ^]^ to map the differentiation and conversion of cell subtypes in each subtype of cell. The top eight genes with the most variation were selected from each cluster to construct the trajectory using the Seurat v3.1.2 function FindVairableFeatures. Dimension‐reduction analysis was performed by DDRTree. The plot_cell_trajectory program was used to visualize the trajectory. For RNA velocity analysis, BAM files containing each cell and reference genome were input into the analysis with velocyto^[^ ^54^ ^]^ in Python with default parameters. To assess consistency, the result was mapped to a UMAP plot via Seurat clustering analysis.

### Single‐Cell Entropy and Cell Cycling Analysis

To assess and score the stemness of cancer cells, the entropy of gene expression based on single‐cell RNA expression profiles was assessed by the SLICE (version 0.99.0) algorithm.^[^ ^55^ ^]^ A SLICE object was created to conduct bootstrap calculation of single‐cell gene entropy values by the getEntropy function after removing ribosomal and ERCC spike‐in genes. The cell cycle phase scores were calculated based on canonical markers using the Seurat program. The G1 phase was defined as noncycling, and G2M and S were defined as cycling states.

### Cellular Communication Analysis

Cell–Cell communication between immune/stromal cells and cancer cells was predicted based on known ligand–receptor pairs by CellphoneDB version 2.1.0.^[^ ^56^ ^]^ In randomized cell identities, the permutation number was set to 1000 when calculating the null distribution of average ligand–receptor pair expression. According to the average log‐transformed gene expression distribution, individual receptor or ligand expression was thresholded across each cell type. The pairs with average log‐transformed expression > 0.1 and *P* value < 0.05 as significant predicted interaction pairs were defined.

### Collection and Process of Bulk Transcriptome Data

Bulk transcriptome data and corresponding clinical information were downloaded from the Gene Expression Omnibus (GEO) database (ID: GSE56493, GSE12276, GSE46141, and GSE173661) as described previously.^[^ ^57^ ^]^ “AnnoProbe” R package was utilized to map the probes. “limma” R package was used to calculate the average values of multiple probes if necessary. These datasets were integrated by “combat” function (“sva” R package).

### Dissection of Tumor Microenvironment Based on Bulk Transcriptome Data

The “xCell” algorithm was used to calculate the tumor microenvironment scores based on bulk transcriptome data.^[^ ^58^ ^]^ Classic immune checkpoint genes were also collected. “ggplot” R package was used to be plotted to visualize the characterization of the tumor microenvironment components, immune checkpoint genes, and immune infiltration score among different metastatic sites.^[^ ^59^ ^]^

### Transwell Assay

MDA‐MB‐231 and MCF‐7 cells were digested and then resuspended. Totally, 5×10^4^ cells were added to the superior chambers (without FBS) with DMSO or ML264. Lower cross‐pore compartment contains 20% FBS. After incubation for 24 hours, the cells were fixed with methanol and stained with crystal violet (0.1%). Then, cells on the top surface of the membrane were wiped off, and cells on the lower surface were imaged and counted as described previously.^[^ ^60^ ^]^ Three random fields were photographed and the number of migrated cells was counted. The experiments were performed in triplicate.

### Colony Formation Assay

MDA‐MB‐231 and MCF‐7 cells (5×10^3^) were seeded in 6‐well plates and treated with DMSO or ML264. After 14 days of cultivation, colonies were fixed in methanol before being stained with crystal violet (0.1%). The colonies in each well were imaged and counted. The experiments were performed in triplicate.

### Wound Healing Assay

Briefly, MDA‐MB‐231 and MCF‐7 cells were seeded in 6‐well plates. After forming the confluent monolayers, cells were scraped with a 200 µL sterile pipette tip to create a linear wound. Then, cells were cultured in no‐serum medium with DMSO or ML264 in the following 24 h. The wound healing process was observed under the inverted microscope at 0 and 24 h period. Wound closure analysis was performed using Image J software. The experiments were performed in triplicate.

### Multiplex Immunofluorescent Staining

Paraffin‐embedded tissues were used to conduct immunofluorescent staining as described previously.^[^ ^61^ ^]^ The paraffin‐embedded sections were dewaxed in xylene, rehydrated with graded ethanol, subjected to endogenous peroxidase activity blockade, and subjected to antigen retrieval at high temperature. The sections were permeabilized in TBST (PBS with 0.5% Triton X‐100) and incubated overnight at 4 °C with the primary antibodies. The primary antibodies were applied sequentially, followed by incubation with the secondary antibody and fluorophore. Primary antibodies included anti‐CK19 (Abcam; ab52625; 1:800), anti‐CD3 (Abcam; ab135372; 1:50), anti‐CD68 (Abcam; ab201340; 1:200), and anti‐*α*‐SMA (Affinity; AF1032; 1:1000).

### Statistical Analysis

Statistical analysis was performed using R software, including two‐sided Student's t test, two‐sided Pearson correlation test, and two‐sided Wilcoxon test. The results are presented as means ± standard deviation. All boxplots indicate median (center), 25th and 75th percentiles (bounds of box), and minimum and maximum (whiskers). Log‐rank test was used in Kaplan‐Meier survival analysis. *P* < 0.05 was considered statistically significant.

## Conflict of Interest

The authors declare no conflict of interest.

## Author Contributions

Y.Z., F.Y., and Y.K. contributed equally to this work. H.T., X.X., Z.S.C., and Y.Z. designed the study. Y.Z., F.Y., Y.K., X.H., X.D., J.X., X.O., S.W., L.W., Y.X., W.T., Y.T., and C.W.W. analyzed and interpreted the data. F.Y., Y.K., and C.S. collected clinical samples. Y.Z., X.D., and J.X. conducted the experiments. Y.Z., F.Y., and Y.K. wrote the manuscript. X.H. and Z.S.C. edited the manuscript. H.T., X.X., and Z.S.C. final reviewed the manuscript. All authors read and approved the final manuscript.

## Supporting information

Supporting Information

## Data Availability

The data that support the findings of this study are available from the corresponding author upon reasonable request.
