# Supplementary material for: The Single‐Cell Landscape of Intratumoral Heterogeneity and The Immunosuppressive Microenvironment in Liver and Brain Metastases of Breast Cancer
Source: Adv Sci (Weinh). 2022 Dec 18;10(5):2203699. doi: 10.1002/advs.202203699 (PMC9929130; doi:10.1002/advs.202203699)
Supplement: Supplementary file 1 — Supporting Information [file ADVS-10-2203699-s001.pdf]

ADVANCED SCIENCE
Open Access

Supporting Information

for *Adv. Sci.*, DOI 10.1002/advs.202203699

The Single-Cell Landscape of Intratumoral Heterogeneity and The Immunosuppressive Microenvironment in Liver and Brain Metastases of Breast Cancer

Yutian Zou, Feng Ye, Yanan Kong, Xiaoqian Hu, Xinpei Deng, Jindong Xie, Cailu Song, Xueqi Ou, Song Wu, Linyu Wu, Yi Xie, Wenwen Tian, Yuhui Tang, Chau-Wei Wong, Zhe-Sheng Chen, Xinhua Xie* and Hailin Tang**

Supporting Information

The single-cell landscape of intratumoral heterogeneity and the immunosuppressive microenvironment in liver and brain metastases of breast cancer

Yutian Zou[1,†]; Feng Ye[1,†]; Yanan Kong[1,†]; Xiaoqian Hu[2]; Xinpei Deng[1]; Jindong Xie[1]; Cailu Song[1]; Xueqi Ou[1]; Song Wu[1]; Linyu Wu[1]; Yi Xie[1]; Wenwen Tian[1]; Yuhui Tang[1]; Chau-Wei Wong[1]; Zhe-Sheng Chen[3,*]; Xinhua Xie[1,*]; Hailin Tang[1,*]

Author Affiliations:

[1] Sun Yat-sen University Cancer Center; State Key Laboratory of Oncology in South China; Collaborative Innovation Center for Cancer Medicine, Guangzhou, China.

[2] School of Biomedical Sciences, Faculty of Medicine, The University of Hong Kong, Hong Kong, China.

[3] College of Pharmacy and Health Sciences, St. John's University, Queens, NY, USA.

*** Corresponding authors:**

Hailin Tang (tanghl@sysucc.org.cn), Xinhua Xie (xiexh@sysucc.org.cn):

Sun Yat-sen University Cancer Center,

State Key Laboratory of Oncology in South China, Collaborative Innovation Center for Cancer Medicine, 651 Dongfeng East Road, Guangzhou, 510060, China.

Zhe-Sheng Chen (chenz@stjohns.edu):

Department of Pharmaceutical Sciences, College of Pharmaceutical Sciences, St. John's University, 8000 Utopia Parkway, Queens, NY 11439, USA.

Supplementary Figures

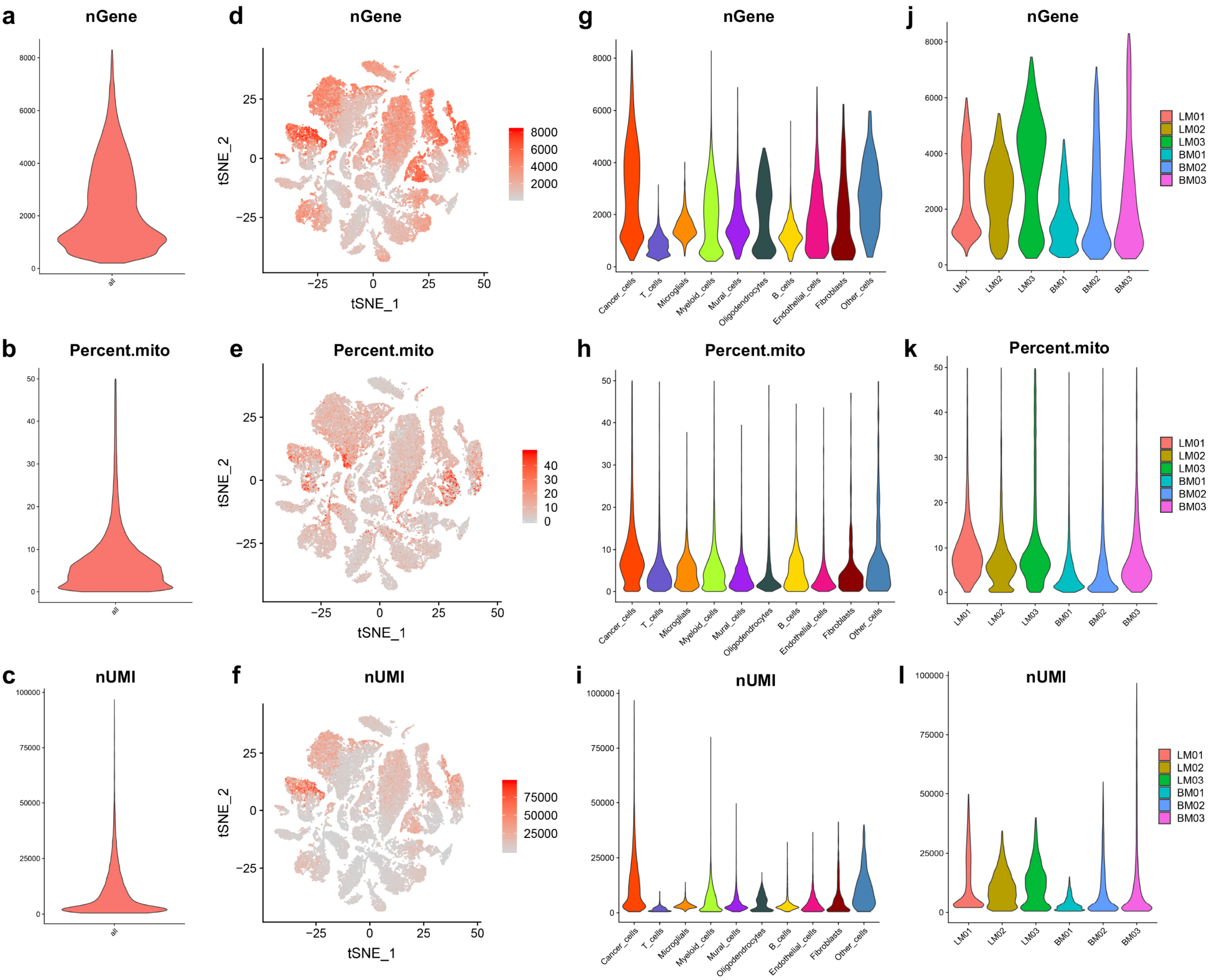

Supplementary Figure 1. Quality control of the single-cell sequencing data.

(a-c) The violin plots showing the number of genes (nGene) detected, percent of mitochondrial derived transcripts (percent.mito), and number of unique molecular identifiers (nUMI). **(d-f)** t-SNE plots of single cells profiled colored by nGene, percent.mito, and nUMI. **(g-i)** The violin plots showing the distribution of nGene, percent.mito, and nUMI in each cell cluster. **(g-i)** The violin plots showing the distribution of nGene, percent.mito, and nUMI in each sample.

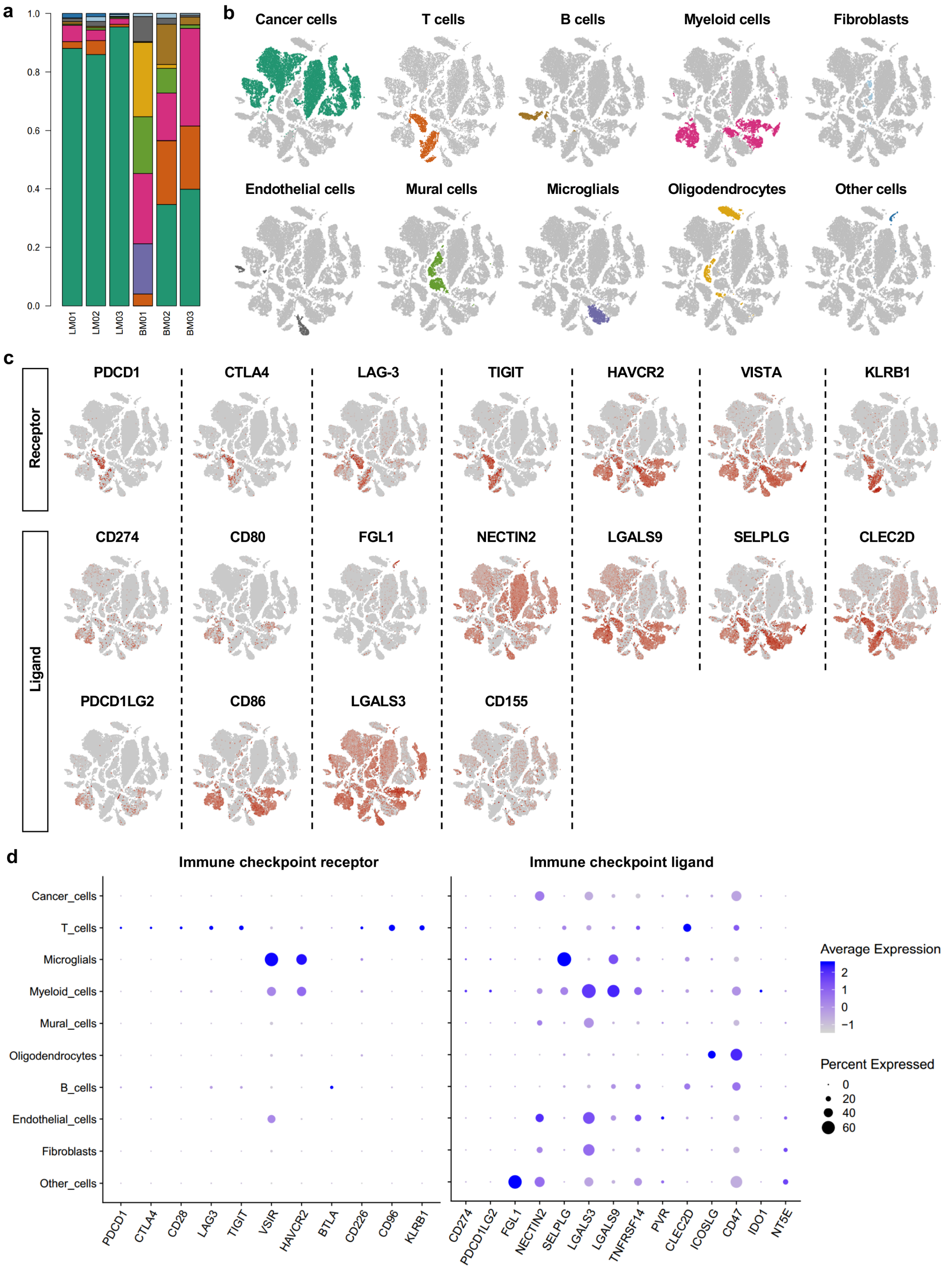

Supplementary Figure 2. Tumor ecosystem of breast cancer liver and brain metastases characterized by single-cell transcriptomic sequencing

(a) The relative proportions of each major cell subtype in different samples. **(b)** Highlight t-SNE plots exhibiting the distribution of each major cell type. **(c)** Feature plots showing the normalized expression of

immune checkpoint genes in each major cell subtype. **(d)** Dot plot showing the expression level of immune checkpoint genes in each major cell type.

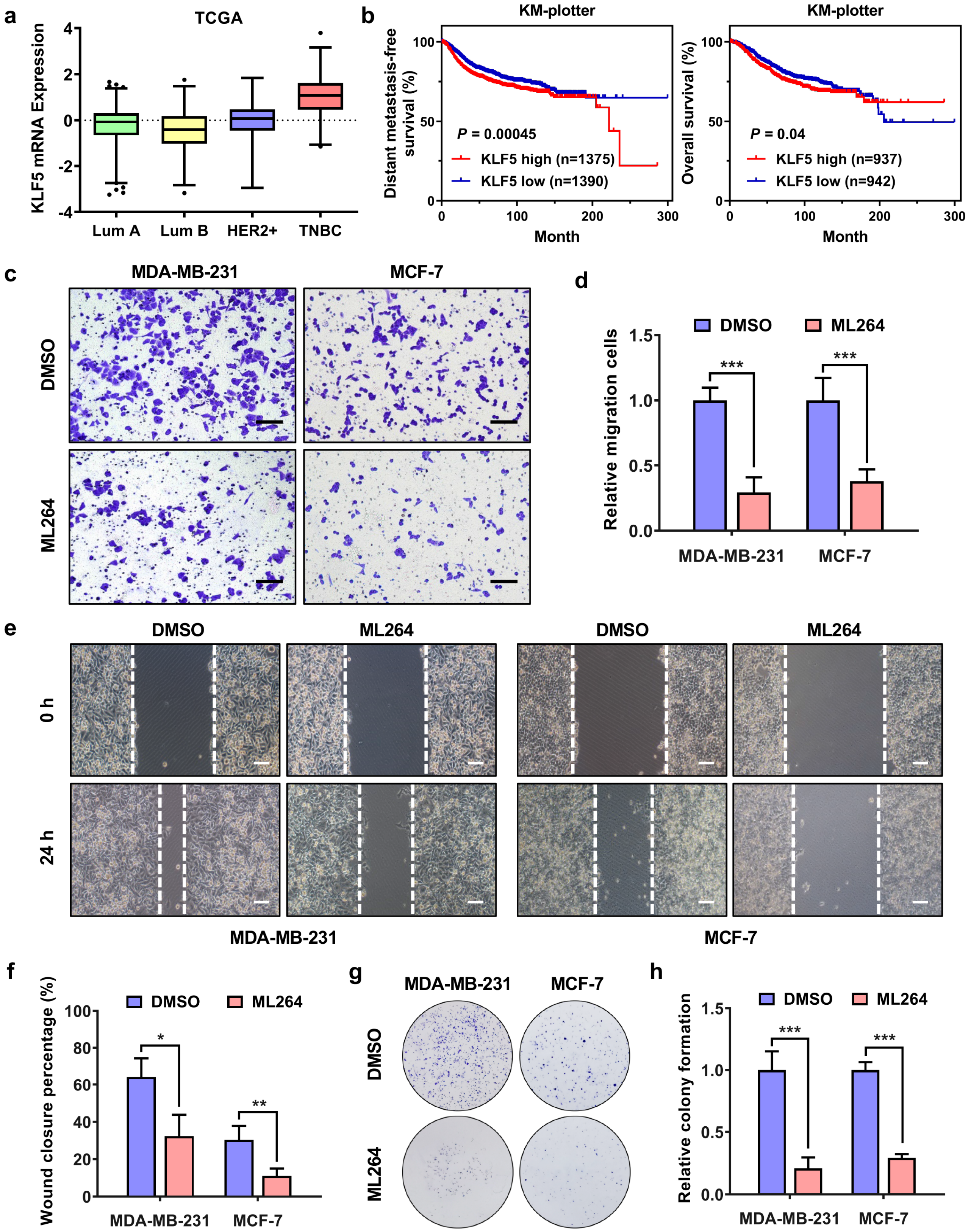

Supplementary Figure 3. Identification and validation of KLF5 as a druggable target for inhibiting breast cancer metastasis.

(a) Expression level of *KLF5* mRNA in different molecular subtypes of breast cancer in TCGA cohort. **(b)** Kaplan-Meier analysis of the distant metastasis-free survival and overall survival of breast cancer patients

with *KLF5* high or low expression in public database cohorts. **(c-d)** Transwell migration assay revealed the effect of KLF5 inhibitor (ML264, 5 μM) in inhibiting the metastatic ability of MDA-MB-231 and MCF-7 breast cancer cells. Representative graphs and quantification are showed. Scale bar = 100 μm. **(e-f)** Wound healing assay revealed the effect of KLF5 inhibitor (ML264, 5 μM) in inhibiting the migration ability of MDA-MB-231 and MCF-7 breast cancer cells. Representative graphs and quantification are showed. Scale bar = 100 μm. **(g-h)** Colony formation assay revealed the effect of KLF5 inhibitor (ML264, 5 μM) in inhibiting the colonize ability of MDA-MB-231 and MCF-7 breast cancer cells. Representative graphs and quantification are showed.

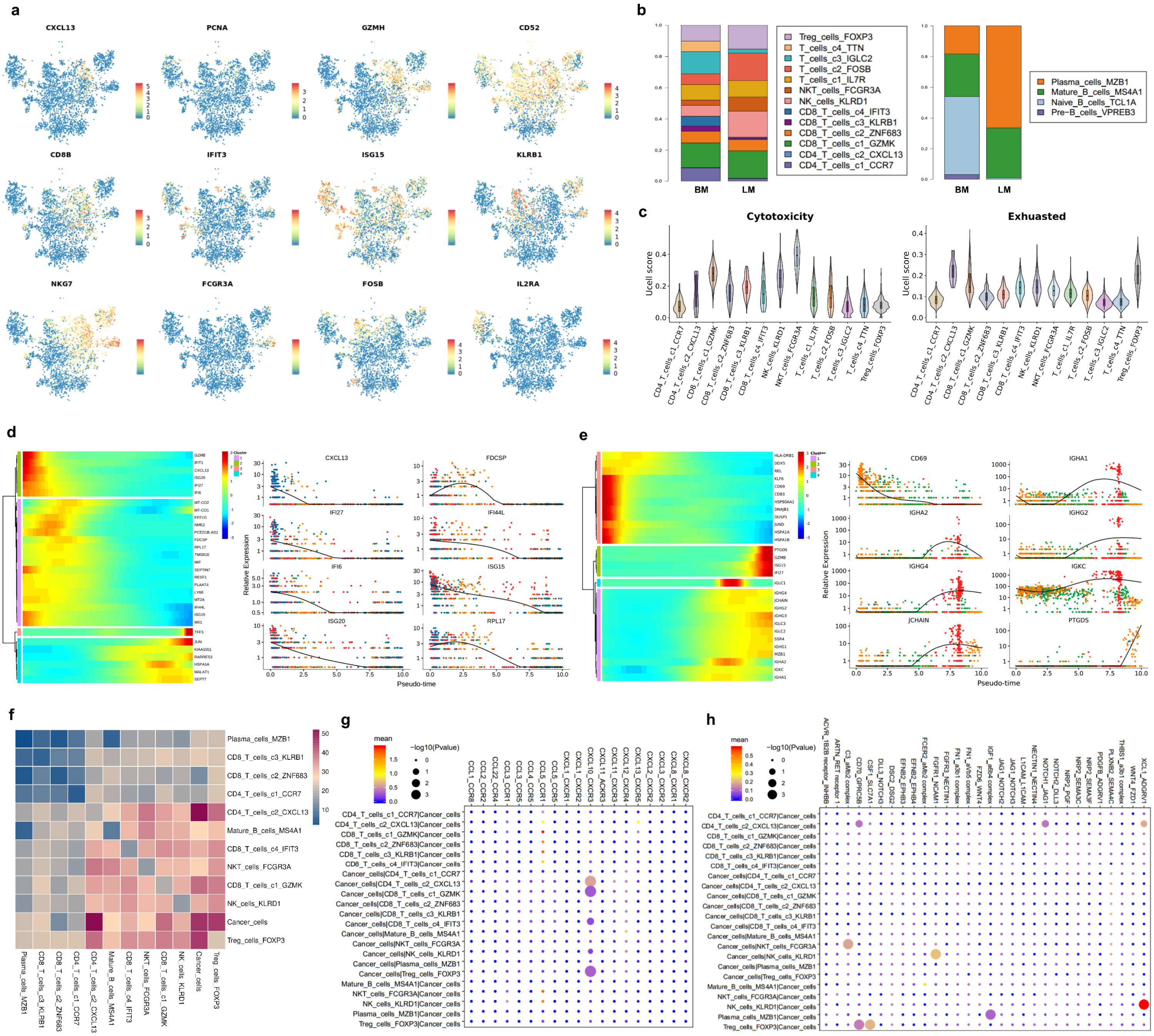

Supplementary Figure 4. Landscape of lymphocytes and innate lymphoid cells in breast cancer liver and brain metastases

(a) Feature plots showing the normalized expression of canonical marker genes in each T cell and NK cell subcluster. **(b)** The relative proportions of each T cell subtype and B cell subtype in liver and brain metastasis of breast cancer. **(c)** Cytotoxicity and dysfunction of each T cell and NK cell cluster was analyzed and quantified by gene signature scores. **(d)** The expression of most varied genes involved in the CD8+ T cell state transition are showed. **(e)** The expression of most varied genes involved in the B cell state transition are showed. **(f)** Heatmap showing the number of cell-cell interactions between lymphocytes and cancer cells, predicted by CellphoneDB 2 method. **(g)** Dot plot showing the ligand-receptor pairs of chemokines between

cancer cells and each lymphocyte cluster, predicted by CellphoneDB 2 method. **(h)** Dot plot showing the other ligand-receptor pairs between cancer cells and each lymphocyte cluster, predicted by CellphoneDB 2 method.

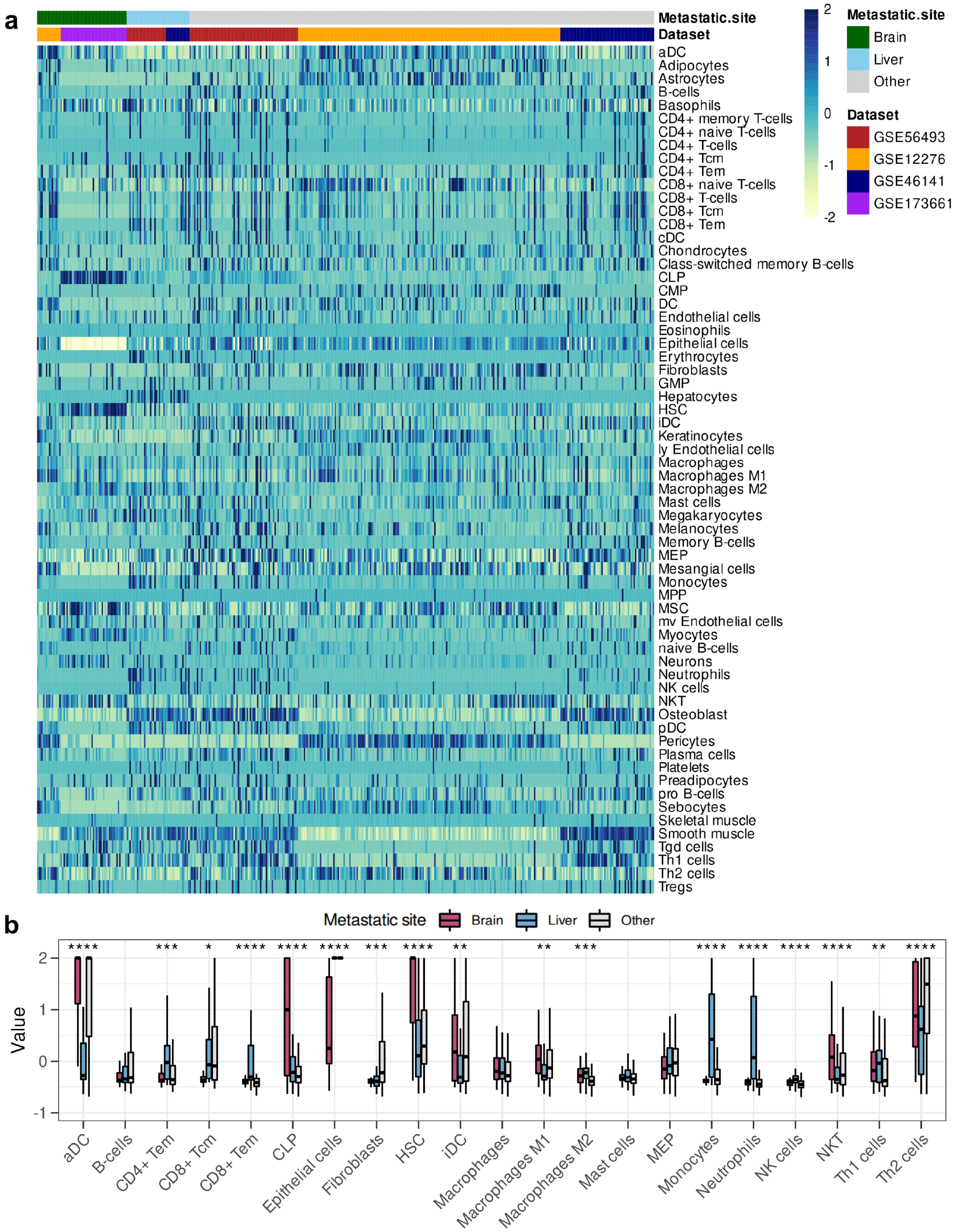

Supplementary Figure 5. Characterization of the tumor microenvironment components among different metastatic sites of breast cancer using bulk transcriptome data.

(a) Heatmap of the tumor microenvironment components calculated by xCell algorithm among different metastatic sites in GEO datasets (GSE56493, GSE12276, GSE46141, and GSE173661). **(b)** Boxplots of the

abundance of immune cells among different metastatic sites in GEO datasets (GSE56493, GSE12276, GSE46141, and GSE173661). **** Means $P < 0.0001$; *** Means $P < 0.001$; ** Means $P < 0.01$; * Means $P < 0.05$.

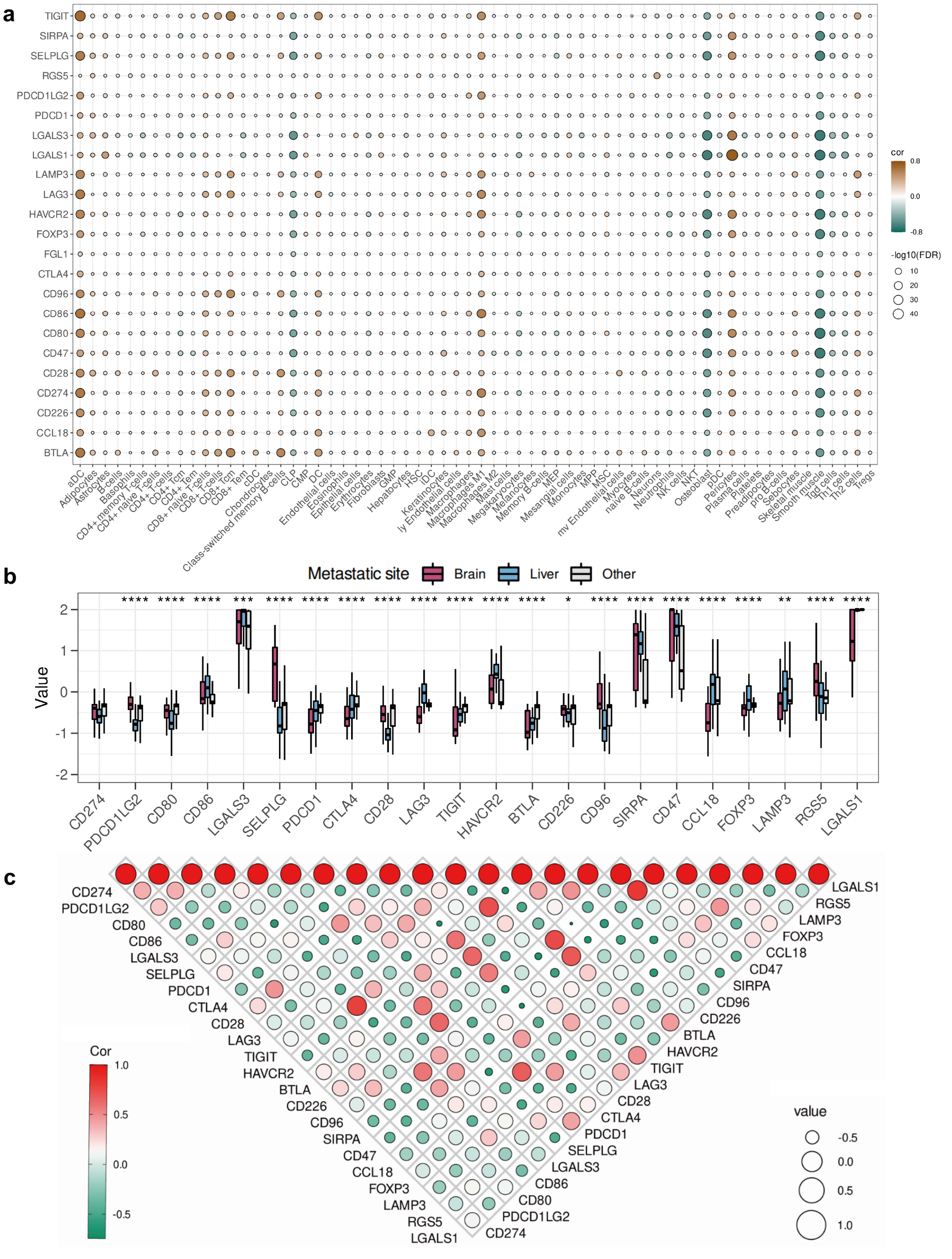

Supplementary Figure 6. Characterization of the immune checkpoint genes and immune cells among different metastatic sites of breast cancer using bulk transcriptome data.

(a) Bubble plot of the relationship between immune checkpoint genes and immune cells in different metastatic

sites of breast cancer. **(b)** Boxplots of the expression of immune checkpoint genes among different metastatic sites of breast cancer in GEO datasets (GSE56493, GSE12276, GSE46141, and GSE173661). **** Means P < 0.0001; *** Means P < 0.001; ** Means P < 0.01; * Means P < 0.05. **(c)** Correlation between the expression of each immune checkpoint.

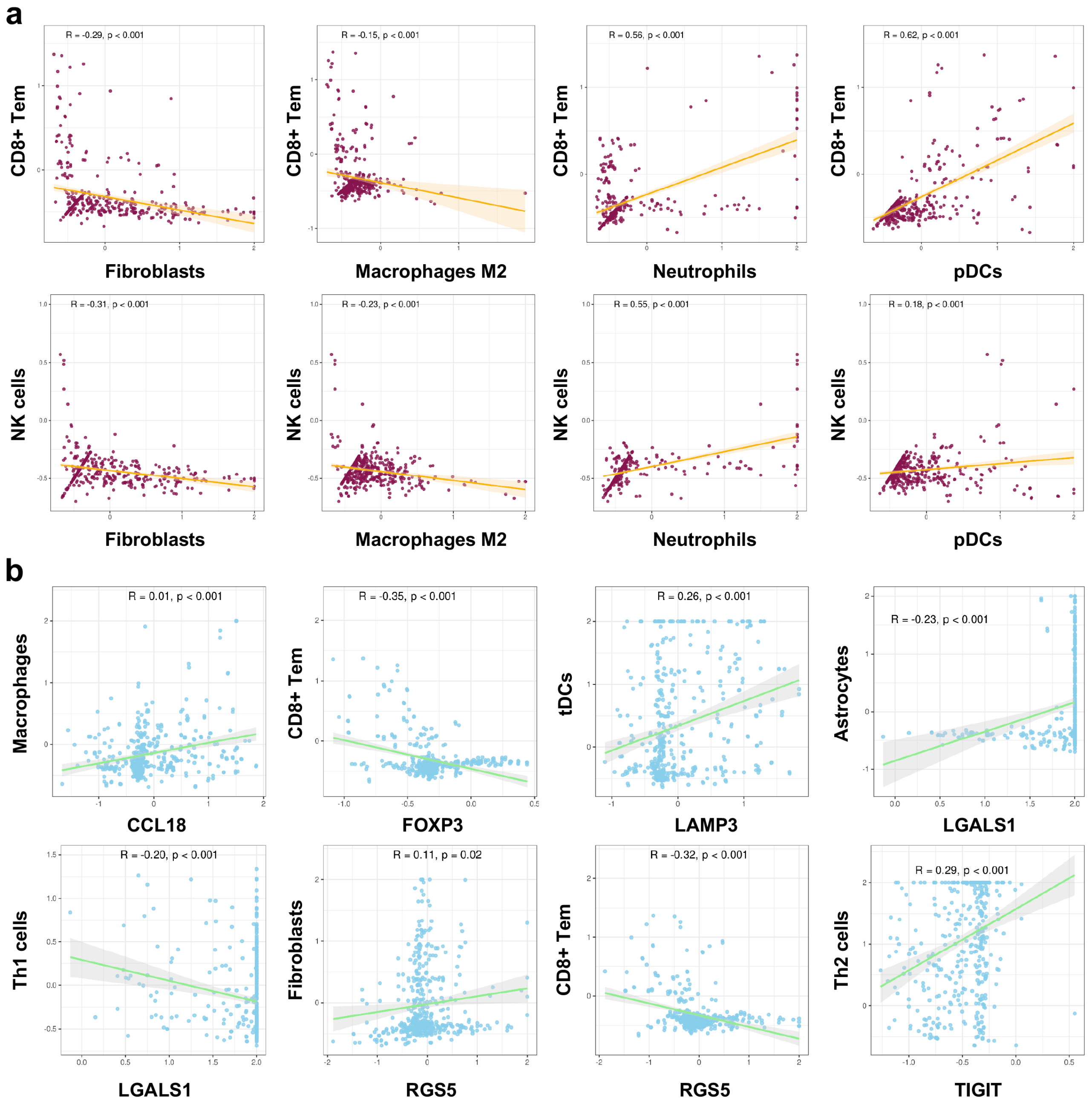

Supplementary Figure 7. Correlation between different tumor microenvironment components and immune checkpoint genes among metastatic breast cancer using bulk transcriptome data.

(a) Correlation between specific immune effector cells (CD8+Tem and NK cell) and other tumor microenvironment cells in metastatic lesions of breast cancer (GEO dataset cohorts were analyzed, including GSE56493, GSE12276, GSE46141, and GSE173661). **(b)** Correlation between tumor microenvironment cells and immune checkpoint genes in metastatic lesions of breast cancer (GEO dataset cohorts were analyzed, including GSE56493, GSE12276, GSE46141, and GSE173661).

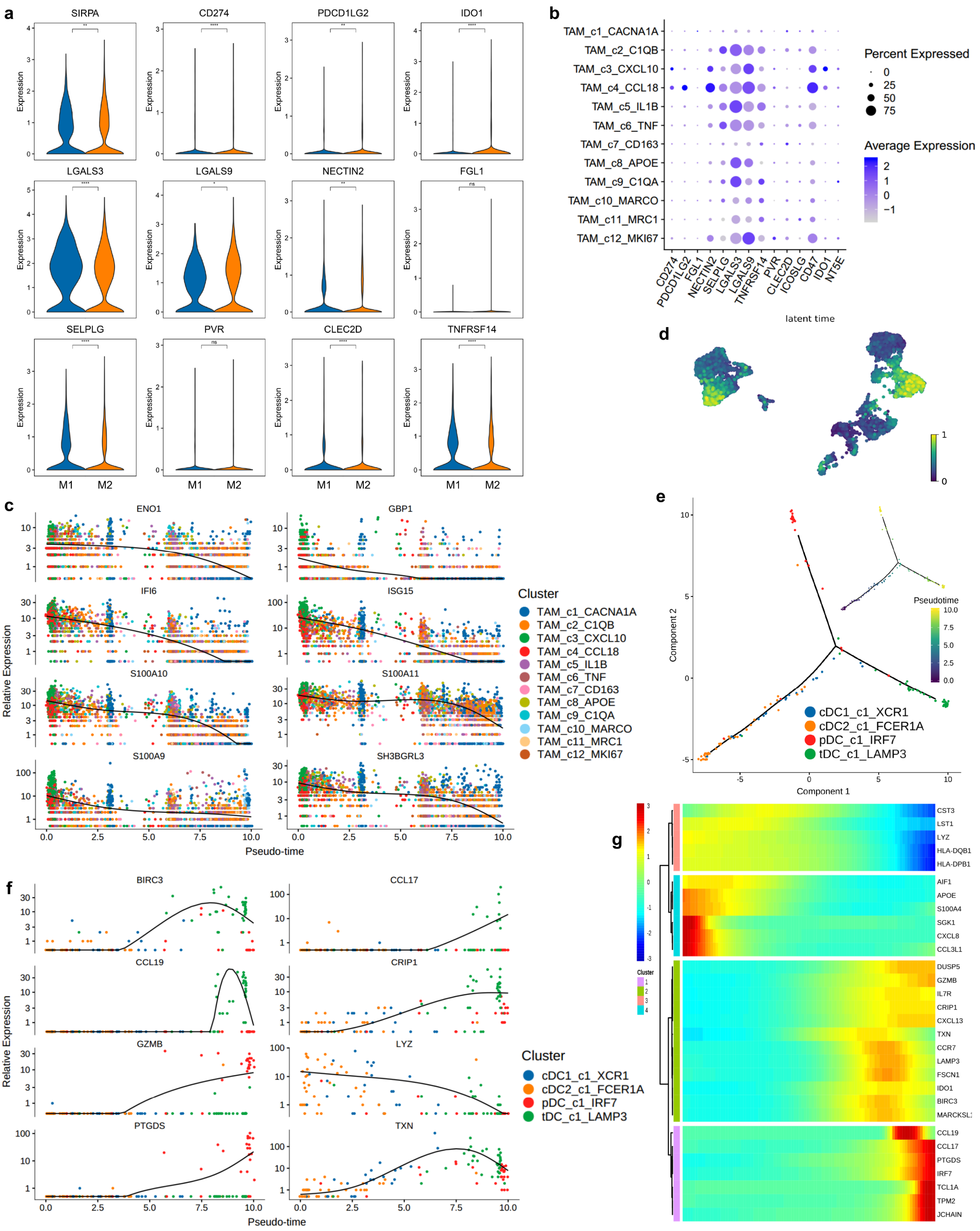

Supplementary Figure 8. Characterization of immunosuppressive myeloid cell in the tumor microenvironment

(a) Violin plot comparing the expression level of phagocytic inhibitory gene (*SIRPA*) and immune checkpoint genes in M1-like and M2-like tumor-associated macrophages (TAMs). **(b)** Dot plot showing the expression

level of immune checkpoint genes in each TAM subclusters. **(c)** The expression of most varied genes involved in the TAM state transition are showed. **(d)** RNA velocity analysis was performed to investigate developmental lineages and cellular dynamics of TAMs. **(e)** Monocle pseudotime trajectory analysis of dendritic cells (DCs) with high variable genes. Each dot on the pseudotime curve represents one single cell, which is colored corresponding to its cluster label. **(f)** The expression of most varied genes involved in the DC state transition are showed. **(g)** The differentially expressed genes along with the DC pseudotime curve is showed in hierarchical heatmap.

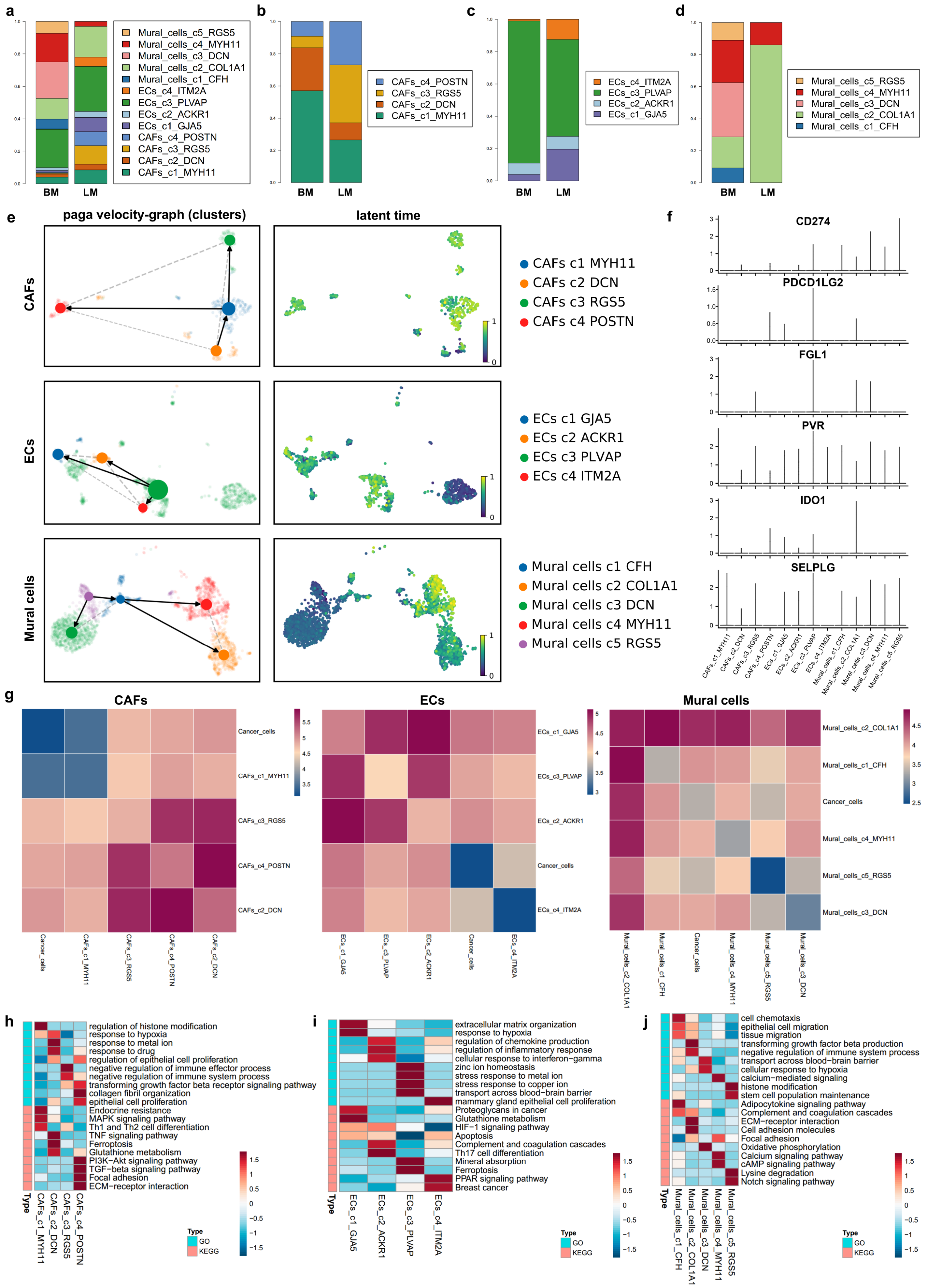

Supplementary Figure 9. Diversity of cancer-associated fibroblasts (CAFs), endothelial cells (ECs) and mural cells (MCs) in the tumor microenvironment

(a) The relative proportions of each stromal cell subcluster in liver and brain metastasis of breast cancer. **(b)**

The relative proportions of cancer-associated fibroblast (CAF) subcluster in liver and brain metastasis of breast cancer. **(c)** The relative proportions of each endothelial cell (EC) subcluster in liver and brain metastasis of breast cancer. **(d)** The relative proportions of each mural cell (MC) subcluster in liver and brain metastasis of breast cancer. **(e)** RNA velocity analysis was performed to investigate developmental lineages and cellular dynamics of CAFs, ECs, and MCs. **(f)** Violin plot showing the expression level of immune checkpoint genes in each stromal cell subtype. **(g)** Heatmap showing the number of cell-cell interactions between CAFs and cancer cells, predicted by CellphoneDB 2 method. **(h)** Heatmap showing the number of cell-cell interactions between ECs and cancer cells. **(i)** Heatmap showing the number of cell-cell interactions between mural cells and cancer cells. **(j-l)** The potential biological functions and relevant signaling pathway of each CAF, EC, and MC subcluster was evaluated by GO and KEGG analysis.

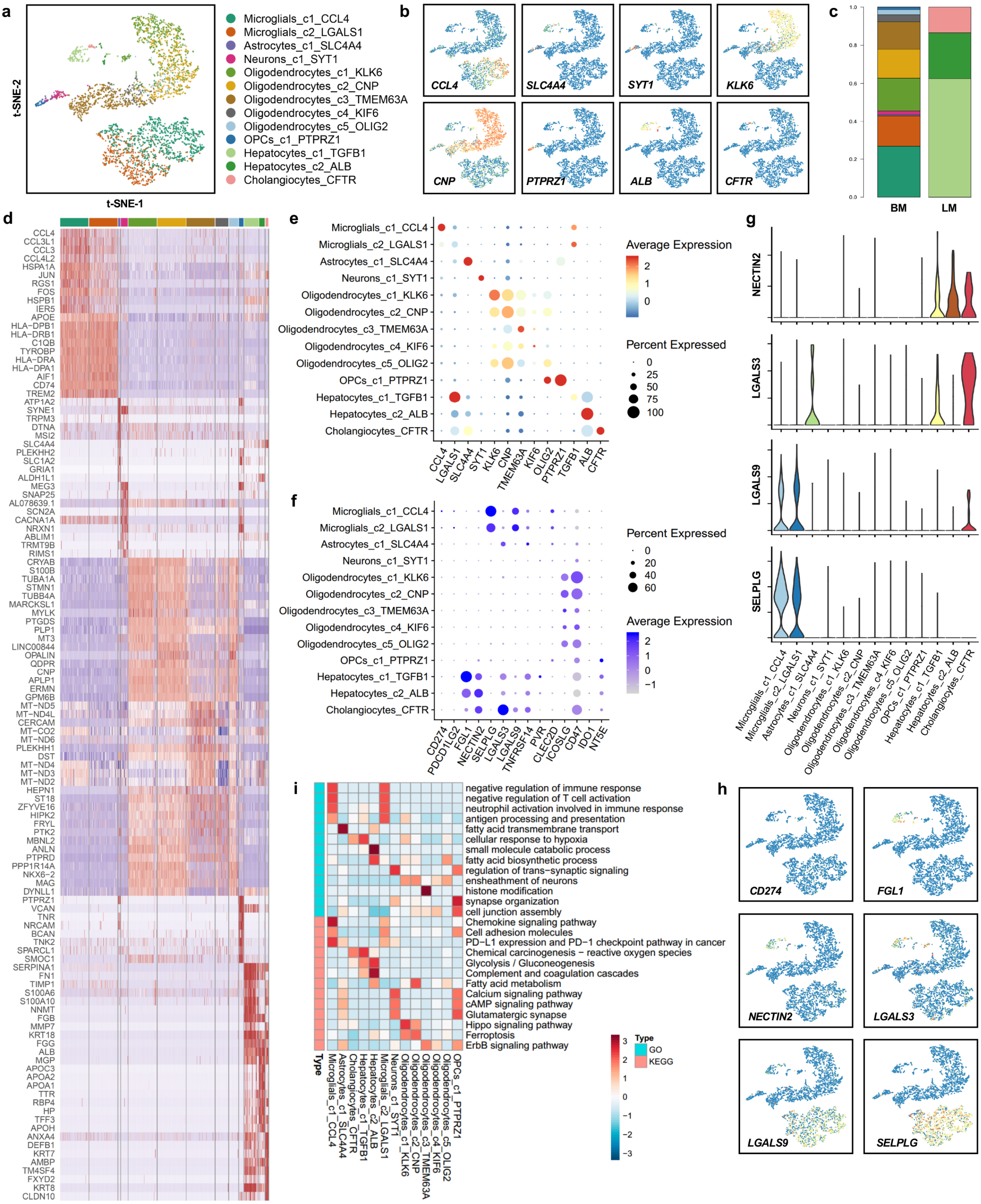

Supplementary Figure 10. Cell clustering and functional annotation of organ-specific resident cells in breast cancer liver and brain metastases

(a) Re-clustering organ-specific resident cells and visualizing the profile of each cell subtype via t-SNE plot.

(b) Feature plots showing the normalized expression of canonical marker genes in each resident cell subcluster.

(c) The relative proportions of each resident cell cluster in liver and brain metastasis of breast cancer. **(d)** The heatmap of the expression level of top 10 differentially expressed genes among thirteen subclusters of resident cells. **(e)** Dot plot shows the expression level of canonical marker gene across all resident cell subtypes. **(f)** Dot plot shows the expression level of immune checkpoint genes across all resident cell subtypes. **(g)** Violin plot showing the expression level of immune checkpoint genes in each resident cell subtype. **(i)** The potential biological functions and relevant signaling pathway of each resident cell subcluster was evaluated by GO and KEGG analysis. **(h)** Feature plots showing the normalized expression of immune checkpoint genes in each resident cell subcluster.

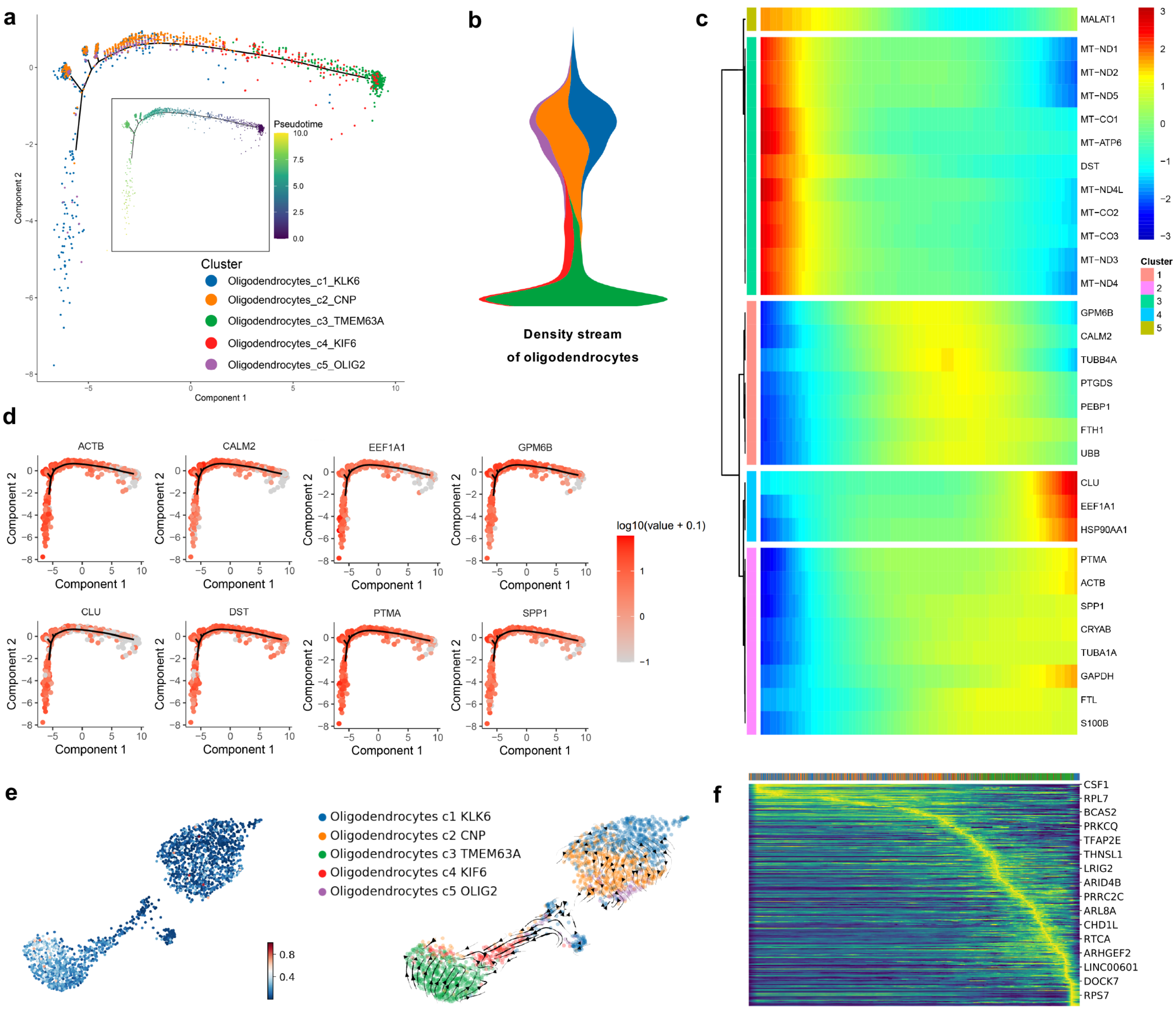

Supplementary Figure 11. Clonal evolution analysis of oligodendrocytes in breast cancer brain metastasis

(a) Monocle pseudotime trajectory analysis of oligodendrocytes with high variable genes. Each dot on the pseudotime curve represents one single cell, which is colored corresponding to its cluster label. **(b)** The density stream of oligodendrocytes clonal evolution. **(c)** The differentially expressed genes along with the oligodendrocyte pseudotime curve is showed in hierarchical heatmap. **(d)** The expression of most varied genes involved in the oligodendrocyte state transition are showed. **(e-f)** RNA velocity analysis was performed to investigate developmental lineages and cellular dynamics of oligodendrocytes.

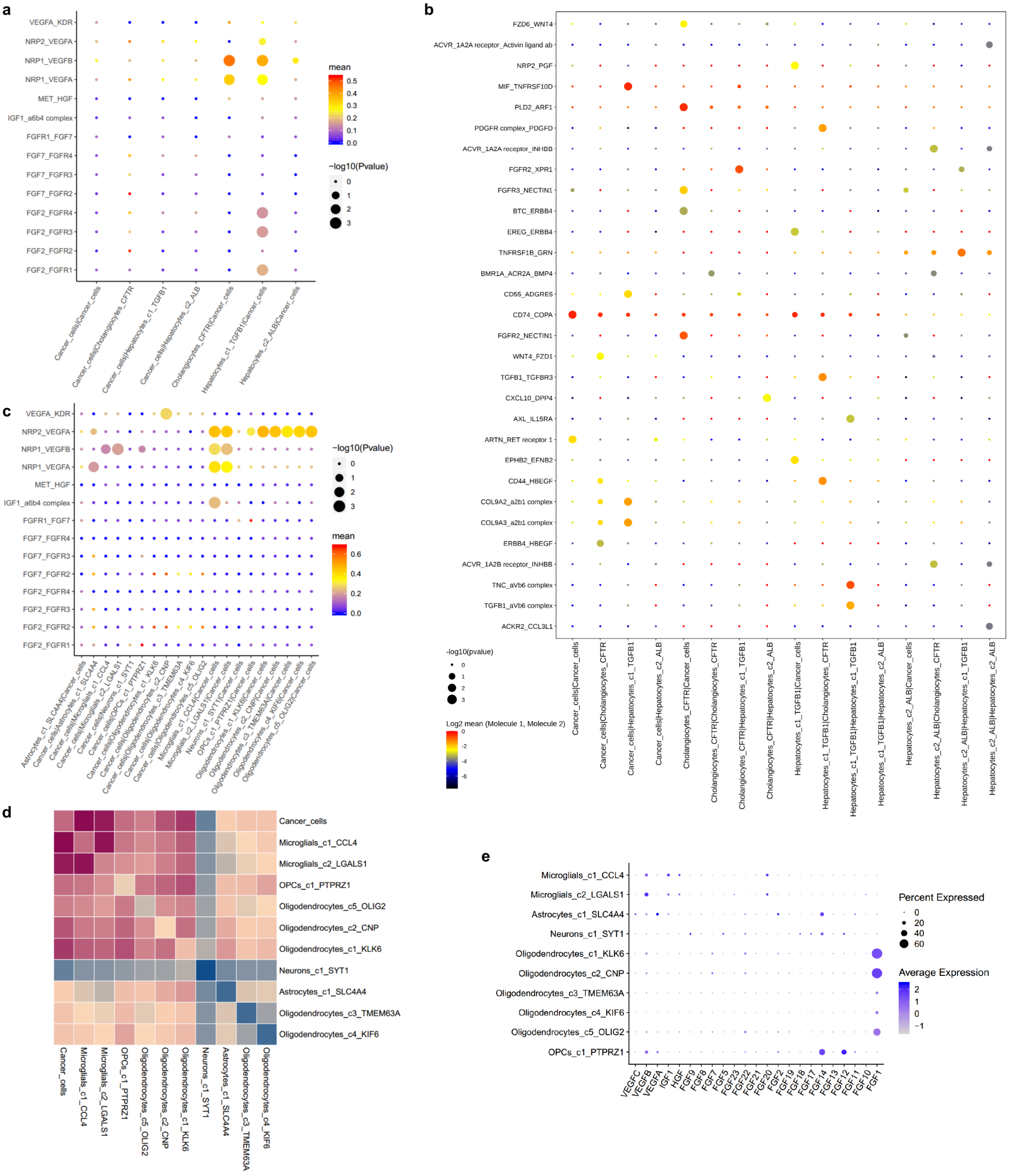

Supplementary Figure 12. Cell-cell communication network between resident cells and cancer cells in breast cancer liver and brain metastasis

(a) Dot plot showing the ligand-receptor pairs of cell growth factors between cancer cells and each resident cell cluster in liver metastasis of breast cancer, predicted by CellphoneDB 2 method. **(b)** Dot plot showing the other significant ligand-receptor pairs between cancer cell and each resident cell cluster in liver metastasis of breast cancer. **(c)** Dot plot showing the ligand-receptor pairs of cell growth factors between cancer cells and each resident cell cluster in brain metastasis of breast cancer. **(d)** Heatmap showing the number of cell-cell interactions between resident cells and cancer cells in brain metastasis of breast cancer, predicted by CellphoneDB 2 method. **(e)** Dot plot shows the expression level of cell growth factor genes across all resident cell subtypes.

Supplementary Table 1: Clinical information for breast cancer patients analyzed by scRNA-seq in this study.

Case ID	Gender	Age	Menstrual status	Metastatic site	Metastasis pattern	Time to metastasis (Month)	Pre-treatment of metastatic disease
LM01	Female	49	Pre-menopause	Liver	Heterochronous	56	/
LM02	Female	51	Pre-menopause	Liver	Heterochronous	48	/
LM03	Female	55	Pre-menopause	Liver	Heterochronous	102	/
BM01	Female	48	Pre-menopause	Brain	Heterochronous	53	Trastuzumab, Pertuzumab, nab-paclitaxel
BM02	Female	54	Post-menopause	Brain	Heterochronous	28	/
BM03	Female	41	Pre-menopause	Brain	Heterochronous	22	/

Supplementary Table 2: Pathological and immunohistochemical information for breast cancer patients analyzed by scRNA-seq in this study.

Case ID	Primary tumor						Metastatic tumor				
	Pathological type	Grade	ER	PR	HER2 (IHC)	Ki67	ER	PR	HER2 (IHC)	HER2 (FISH)	Ki67
LM01	IDC	2	90%+	90%+	3+	40%+	99%+	90%+	3+	/	50%+
LM02	IDC	3	0	0	3+	20%+	95%+	60%+	2+	Non-Amplified	35%+
LM03	IDC	3	0	0	0	30%+	0	0	0	/	85%+
BM01	IDC	3	50%+	20%+	3+	5%+	/	/	/	/	/
BM02	IDC	3	90%+	0	3+	20%+	0	0	3+	/	70%+
BM03	IDC	3	0	0	0	80%+	0	0	0	/	60%+
